# American foulbrood in a honeybee colony: spore-symptom relationship and feedbacks

**DOI:** 10.1186/s12898-020-00283-w

**Published:** 2020-03-06

**Authors:** Jörg G. Stephan, Joachim R. de Miranda, Eva Forsgren

**Affiliations:** 1 Department of Ecology, Swedish University of Agricultural Sciences, 750 07 Uppsala, Sweden; 2 Swedish Species Information Centre, Swedish University of Agricultural Sciences, 750 07 Uppsala, Sweden

**Keywords:** *Apis mellifera*, *Paenibacillus larvae*, Group size, Enzootic disease, Host–pathogen dynamics, Social immunity, MCMC, Detection, SIR-model, Diagnosis, Host density

## Abstract

**Background:**

The most severe bacterial disease of honeybees is American foulbrood (AFB). The epidemiology of AFB is driven by the extreme spore resilience, the difficulty of bees to remove these spores, and the considerable incidence of undetected spore-producing colonies. The honeybee collective defence mechanisms and their feedback on colony development, which involves a division of labour at multiple levels of colony organization, are difficult to model. To better predict disease outbreaks we need to understand the feedback between colony development and disease progression within the colony. We therefore developed Bayesian models with data from forty AFB-diseased colonies monitored over an entire foraging season to (i) investigate the relationship between spore production and symptoms, (ii) disentangle the feedback loops between AFB epidemiology and natural colony development, and (iii) discuss whether larger insect societies promote or limit within-colony disease transmission.

**Results:**

Rather than identifying a fixed spore count threshold for clinical symptoms, we estimated the probabilities around the relationship between spore counts and symptoms, taking into account modulators such as brood amount/number of bees and time post infection. We identified a decrease over time in the bees-to-brood ratio related to disease development, which should ultimately induce colony collapse. Lastly, two contrasting theories predict that larger colonies could promote either higher (classical epidemiological SIR-model) or lower (increasing spatial nest segregation and more effective pathogen removal) disease prevalence.

**Conclusions:**

AFB followed the predictions of the SIR-model, partly because disease prevalence and brood removal are decoupled, with worker bees acting more as disease vectors, infecting new brood, than as agents of social immunity, by removing infected brood. We therefore established a direct link between disease prevalence and social group size for a eusocial insect. We furthermore provide a probabilistic description of the relationship between AFB spore counts and symptoms, and how disease development and colony strength over a season modulate this relationship. These results help to better understand disease development within honeybee colonies, provide important estimates for further epidemiological modelling, and gained important insights into the optimal sampling strategy for practical beekeeping and honeybee research.

## Background

Honeybees are important pollinators in agricultural [1] and natural habitats [2]. The demand for managed pollinators in agriculture has steadily increased during the past decades due to changing diets and an alarming decrease in natural pollinators in cultivated landscapes [3]. At the same time, beekeepers worldwide are experiencing increased winter and seasonal colony losses [4–7]. Such losses stem from a combination of parasites and diseases, poor nutrition, inadequate beekeeping management practices and pesticide exposure; both individually and synergistically [8–16].

One of the major threats to colony health and beekeeping viability is American foulbrood (AFB); a contagious, lethal bacterial disease of honeybee brood that is widely distributed across the world. The disease causes great economic losses during outbreaks due to reduced productivity and material turnover [17–20]. American foulbrood is caused by *Paenibacillus larvae*, a spore-forming bacterium that produces extremely resilient spores which can remain viable for decades [21]. Within a colony, *P. larvae* spores are spread by nurse bees performing in-hive tasks, such as cleaning, but especially through the feeding of young larvae with spore-contaminated food [22]. Billions of spores are produced in the dying larvae [20]. The dried larval remains (scales) are difficult to remove by workers and are a continuous source of infection for new brood. The lethality and epidemiology of AFB are driven by the resilience of the spores and the fact that the removal of diseased brood, a communal bee hygienic behaviour [23, 24], is not sufficient to remove this source of infection [20, 25]. The spores are distributed between colonies by swarming, robbing and in particular by beekeepers moving contaminated material between colonies [26, 27].

One major problem for the control of American foulbrood is that even though clinical symptoms are highly characteristic for the disease, they tend to appear late during the epidemic, when the colony’s hygienic behaviour to remove infected larvae before they produce spores, can no longer keep up with the epidemic. Estimations have been made that as much as 25% of spore-producing colonies remain undetected [28]. Infections are therefore enzootic, since they remain in the population without external inputs [29], and occult, since they are present but largely visually undetected [27]. Colonies can produce large amounts of infectious *P. larvae* spores with relatively few cases of symptomatic brood, thus escaping detection during routine beekeeper inspections while continuing to be a source of infection both within a beekeeping operation, between beekeepers (through sale of bees and equipment) and to feral and managed colonies within flight range through drifting and robbing [30].

One way to address the risk of pre-clinical infectious colonies for epidemic spread at multiple scales (local, regional, national) is to determine this risk directly from *P. larvae* spore levels in material sampled from the colony, thereby unambiguously identifying all infectious colonies rather than just those presenting detectable symptoms. It has been shown that adult bees provide most reliable samples for relating *P. larvae* spore levels to AFB symptoms, superior to either colony debris or honey samples [28, 31–33]. The spore load of individual bees is positively correlated to the likelihood of clinical symptoms [31, 34]. Since the spores are heavily concentrated in the brood frames and hive material, beekeepers can remove much of the colony-level spore burden by shaking the adult bees and queen into new, clean hives and frames with fresh wax foundation [35]. Attempts have been made previously to predict clinical symptoms from the number of spores in a colony [36, 37]. However these attempts lacked two important elements for improving the reliability of such a calibration curve, namely an estimation of uncertainty and the usage of the recommended standardised grading of the severity of AFB symptoms.

We therefore included standardised AFB symptom grading scales [38] and multilevel Bayesian linear models to rectify these deficiencies, in order to identify more reliably the probability of AFB symptoms given a particular spore count. The results obtained are therefore directly applicable to practical beekeeping, as well as to research and epidemiological modelling. For example, the results can be used to parameterize other Bayesian models, by using the posterior probability predicted by these models as prior probability estimates for other Bayesian analyses, *e.g.* for predicting or analysing AFB transmission, infectivity, epidemiology, or symptoms in various actual or theoretical scenarios. Predicting AFB symptoms from spore counts follows a causational logic. However, from a practical perspective it would also be useful to explore the reverse relationship, *i.e.* to predict the colony spore levels from observed symptoms since the primary data obtained from colony inspections is the presence and severity of symptoms, which are then followed up with laboratory spore analyses of adult bee samples. This furthermore also serves as a quality control of the standardised AFB symptom grading system.

Our second objective was to disentangle the natural colony development and the colony-level disease development over a season. While both the development of *P. larvae* infection in larvae [20], and AFB disease epidemiology between colonies using colony infection data [18, 27] have been described, the factors shaping AFB virulence at the colony level are still largely unknown. We focused on the onset and the development of the disease, which are the most relevant disease stages from a practical as well as an epidemiological perspective. AFB can kill a colony within a single season, which in temperate regions of the northern hemisphere ranges from the beginning of April to end of September. Although one previous attempt at modelling AFB development suggests that the onset of symptoms is rather sudden [39], there has been no controlled study to evaluate the time course of AFB progression during a full season. In addition, honeybee colonies are complex entities (super-organisms) where most of the colony dynamics are driven by social interactions and decision making between its members, based on sensory input from within the colony and the environment, which makes it very difficult to predict the course of any disease [26]. Particularly collective defence components, such as hygienic behaviour, and its feedback on colony development are difficult to model reliably, since it involves both brood and adult bees and affects role allocation and decision making at multiple levels in colony organization [24, 40].

Our last objective concerned whether larger honeybee colonies limit or accelerate the epidemiology of AFB. For example colonies that have naturally adapted to survive uncontrolled *Varroa destructor* infestation display unique and characteristic colony development traits, including smaller overall size, reduced drone brood production and lower brood-to-adult bee ratios, all of which are predicted to limit the reproductive potential for this mite [41]. The classical deterministic epidemiological model, based on Susceptible, Infected and Resistant Hosts (the SIR-model [42]) would predict that larger colonies (more brood) should increase the spread of disease. However, living in social groups is clearly beneficial with regard to predation risk [43] and social hygiene, which may outweigh the higher infection risks [44]. For eusocial insect societies in particular, larger colonies may be more able than smaller colonies to deploy counter measures to epidemic disease spread, such as removing diseased brood (hygienic behaviour) [45]. Additionally, the increased spatial separation in larger colonies may delay the spread of infection [40]. Considerable research has been conducted to understand how larger social groups cope with higher infection risk, and factors such as genetic diversity [46], group size [47, 48] and the structure of the social network [49] have been shown to be important for affecting the colony-level effects of infection and disease. Most of these studies aim at the mechanisms social animals use to minimize infection risk (*e.g.* the ability to detect and remove fungal diseases [50] or the effectiveness of cuticular antimicrobial defences in relation to colony size [51]). However, such an approach is contingent on the assumptions related to these biological mechanisms. Here we approach the relationships between disease severity and colony size directly, and therefore independent of mechanistic assumptions. For example the variation in the number of susceptible hosts (*i.e.* the amount of larval brood, for AFB) over a season depends on many intrinsic and external factors [52, 53]. Since American foulbrood kills the brood, a shift in the brood-to-worker ratio can be expected, but it is unclear whether larger honeybee colonies are better than smaller colonies at handling such a shift. In this study we will therefore attempt to describe how AFB affects colony strength parameters (amount of brood and number of adult bees) and how these parameters feed back to the epidemiology in the colony, in order to understand the relevance of colony size itself, independent of colony-level mechanisms, on disease spread.

## Results

**Fig. 1:**
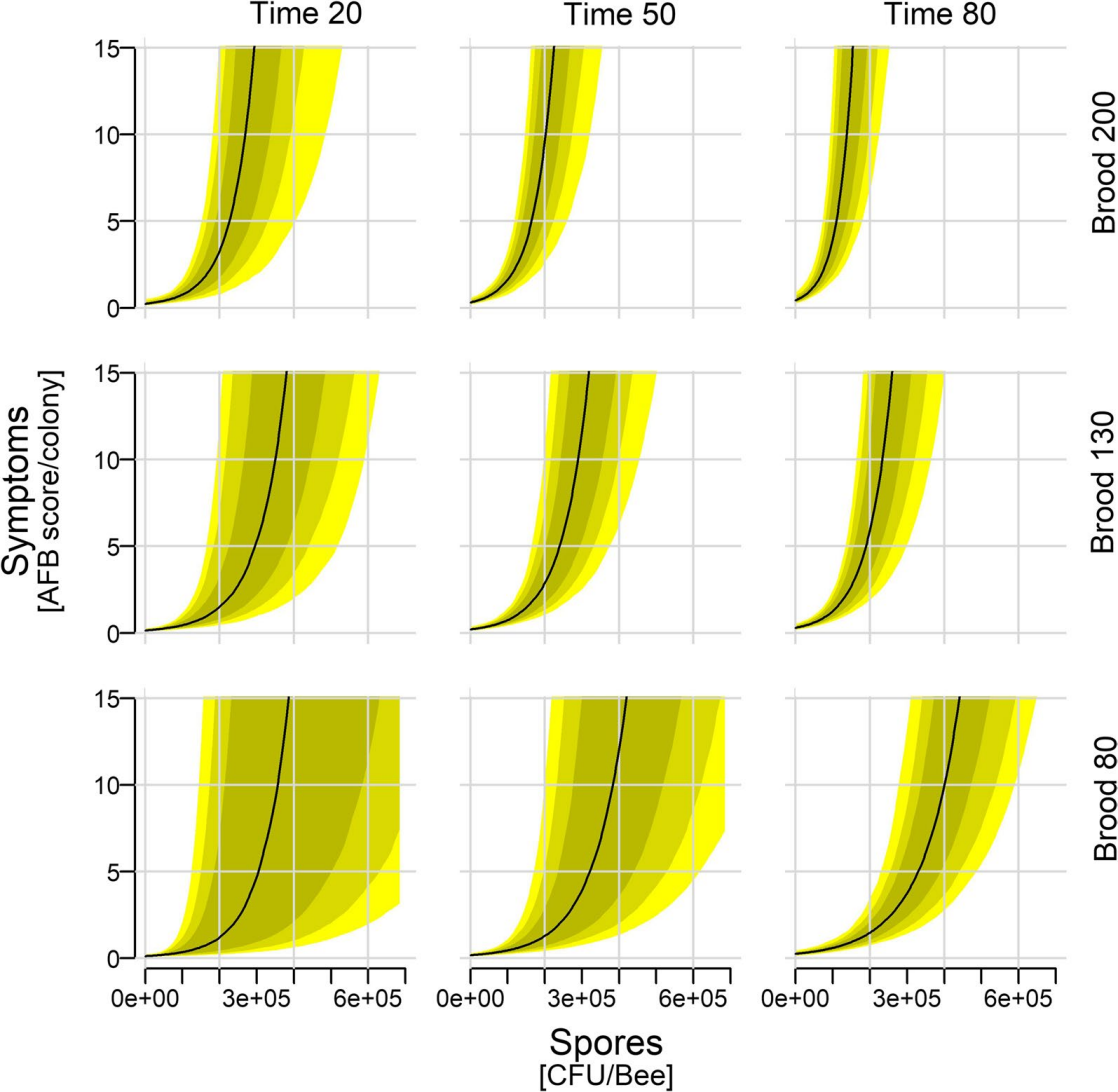
Clinical symptoms depending on spore counts, time of the season, and brood size within the colony. Shown are median (with 97, 89, and 67% credible intervals) posterior distributions along the full range of observed spore counts. The remaining continuous predictors are held approximately at their mean (brood: 132.6; time: 48.4), their 1st quantile (brood: 78; time: 21), and their 3rd quantile (brood: 191; time: 79)

The results describe the importance of a particular predictor for predicting a response variable, the direction of the predicted effect on the response variable, and the extent to which the primary relationship is modulated by secondary and tertiary predictors. Within each figure, the central subfigure shows the relationship between the primary predictor and the response variable if the influence of the other two secondary predictors (top and right) is neutral. Because these secondary predictors mostly co-vary (*i.e.* Fig. 1: Brood 80 at Time 20 is more likely than Brood 200 at Time 20) the three subfigures on the leading diagonal are the most likely outcomes, while the remaining six subfigures are primarily for explaining and understanding the interactions. A summary of the original data can be found in Additional file 1: (Figure S1 and Table S1). Throughout the results variables have capital letters.

### Predicting clinical symptoms from spore counts

All response variables were relevant for predicting Symptoms (Table 2: P[effect > 0] = 100% for all predictors). Spores were a strong predictor of the Symptoms in all four models, both directly and through its interaction with the other variables (Table 1: M1–M4), and because subtracting the effect of secondary predictors from the Spores predictor left a high probability of an effect size larger than zero (Table 2: Spores-Brood P[effect > 0] = 91.3%; Spores-Time P[effect > 0] = 94.3%). Brood and Time were equally predictive for Symptoms (Table 2: Brood-Time P[effect > 0] = 55.6%), though much less so than Spores. The Symptoms increased first slightly and then strongly with increasing Spores, regardless of the time and brood (Fig. 1: Time 50/Brood 130). Symptoms increased over Time, both in absolute terms and in relation to a given spore count level (see also Additional file 2: Figure S2), and Symptoms also increased with increasing Brood (see also Additional file 2: Figure S3).

**Table 1:** Models used to average posteriors by multiplication with the model weight

Response	Model	Explanatory	pWAIC	WAIC	SE	weight
Symptoms	M1	Spores, Time, Brood Spores × Time Spores × Brood Time × Brood Spores × Time × Brood	62.6	481.6	34.12	0.51
	M2	Spores, Time, Brood Spores × Brood	62.4	483.3	34.22	0.23
	M3	Spores, Time, Brood Spores × Brood, Time × Brood	63.1	483.8	34.18	0.18
	M4	Spores, Time, Brood Spores × Time, Spores × Brood Time × Brood	63.3	485.4	34.42	0.08
Spores	M5	Symptoms, Time, Bees Symptoms × Time	118.4	2330.6	59.81	0.50
	M6	Symptoms, Time, Bees Symptoms × Time Symptoms × Bees, Time × Bees Symptoms × Time × Bees	118.7	2332.4	59.23	0.20
	M7	Symptoms, Time, Bees Time × Bees	118.6	2332.7	59.56	0.18
	M8	Symptoms, Time, Bees Symptoms × Bees, Time × Bees	119.3	2333.4	59.51	0.12
Bees	M9	Spores, Time, Brood Spores × Time, Spores × Brood, Time × Brood Spores × Time × Brood	78.2	1453.5	30.69	0.54
	M10	Spores, Time, Brood Spores × Time, Time × Brood	81.0	1454.6	28.60	0.30
	M11	Spores, Time, Brood Spores × Brood	80.3	1457.2	29.16	0.08
	M12	Spores, Time, Brood Spores × Time, Spores × Brood	78.4	1457.4	30.95	0.08
Brood	M13	Symptoms, Time, Bees Symptoms × Bees	141.6	1950.5	15.27	0.997
	M14	Symptoms, Time, Bees Symptoms × Time	144.8	1962.8	12.60	0.002
	M15	Symptoms, Time, Bees Symptoms × Time, Symptoms × Bees	146.2	1965.3	12.34	0.0006
	M16	Symptoms, Time, Bees Symptoms × Time, Time × Bees	146.6	1966.0	13.00	0.0004

Shown are effective Number of Parameters (pWAIC), Widely Applicable Information Criterion (WAIC), standard error of WAIC estimate (SE), and Akaike weight based on WAIC (weight). Remaining models (with different combinations of interactions; not shown) received lower weights and were not used for model averaging

**Table 2:** Posterior distributions for the main parameters on original scale

Response	Parameter	Posterior distribution	P[effect > 0]
Symptoms	Intercept	0.29 ± 0.06 (0.16, 0.43)	100
	Spores	2.13 ± 0.33 (1.47, 2.91)	100
	Time	1.58 ± 0.15 (1.26, 1.91)	100
	Brood	1.61 ± 0.19 (1.28, 2.10)	100
	*Spores*-*Brood*	*0.51 *±* 0.40 (*− *0.26, 1.45)*	*91.3*
	*Brood*-*Time*	*0.03 *±* 0.22 (*− *0.46, 0.52)*	*55.6*
	*Spores*-*Time*	*0.55 *±* 0.39 (*− *0.24, 1.4)*	*94.3*
	*Spores *=* 0*	*0.22 *±* 0.05 (0.12, 0.34)*	*100*
Spores	Intercept	286.08 ± 65.68 (159.83, 432.0)	100
	Symptoms	3.51 ± 0.68 (2.20, 5.06)	100
	Time	0.63 ± 0.08 (0.46, 0.81)	100
	Bees	1.08 ± 0.15 (0.78, 1.46)	100
	*Symptoms*-*Bees*	*2.43 *±* 0.74 (0.97, 4.17)*	*100*
	*Bee*-*time*	*0.45 *±* 0.19 (0.04, 0.87)*	*99.5*
	*Symptoms*-*Time*	*2.88 *±* 0.71 (1.57, 4.49)*	*100*
	*Symptoms *=* 0*	*158.26 *±* 36.58 (92.63, 241.76)*	*100*
	*Symptoms *=* 1*	*228.13 *±* 53.04 (123.54, 349.12)*	*100*
Bees	Intercept	17.27 ± 0.40 (16.41, 18.08)	100
	Spores	1.04 ± 0.02 (0.99, 1.10)	100
	Time	1.13 ± 0.01 (1.10, 1.17)	100
	Brood	1.25 ± 0.01 (1.21, 1.29)	100
	*Brood*-*Spores*	*0.20 *±* 0.03 (0.13,0.29)*	*100*
	*Brood*-*Time*	*0.11 *±* 0.02 (0.06, 0.15)*	*100*
	*Time*-*Spores*	*0.09 *±* 0.03 (0.02,0.166)*	*99.7*
Brood	Intercept	88.11 ± 17.36 (58.69, 127.88)	100
	Symptoms	1.30 ± 0.10 (1.10, 1.59)	100
	Time	0.52 ± 0.03 (0.44, 0.61)	100
	Bees	1.89 ± 0.15 (1.60, 2.29)	100
	*Bees*-*Symptoms*	*0.58 *±* 0.19 (0.16, 1.02)*	*99.9*
	*Bee*-*Time*	1.37 ± 0.16 (0.97, 1.71)	100
	*Symptoms*-*Time*	0.78 ± 0.12 (0.53, 1.07)	100

Show are mean ± standard deviation (with 97% credibility intervals) of the main effects and the effect probability. Posteriors are weighted based on the four selected models (except for brood as response variable, see Table 1 and text). Italic rows specify the importance of one predictor in relation to another (posterior distribution of one parameter minus the other). We also show posteriors for specific values (*Spores/Symptoms *=* 0*; *Symptoms *=* 1*; see text for further explanations)

Brood was a stronger modulator of the Spores-Symptoms relationship than Time since the Spores × Brood interaction received more weight than the Spores × Time interaction (Table 1: M2 includes Spores × Brood and not Spores × Time). This is also illustrated by a stronger change of the Spores-Symptoms relationship along Brood than along Time (Fig. 1).

Lastly we calculated the probability of encountering Symptoms if no Spores are detected in an adult bee sample (*Spores *=* 0*), which resulted in a probability around 0.22.

### Predicting spore counts from clinical Symptoms

All response variables were relevant for predicting Spores (Table 2). Symptoms were the strongest predictors of Spores, both directly and through its many significant interactions with other predictors; three out of the four selected models (Table 1), and since subtracting the effects of secondary predictors from symptoms left a high probability of an effect size larger than zero (Table 2). Bees seemed more important than Time for predicting Spores (Table 2).

**Fig. 2:**
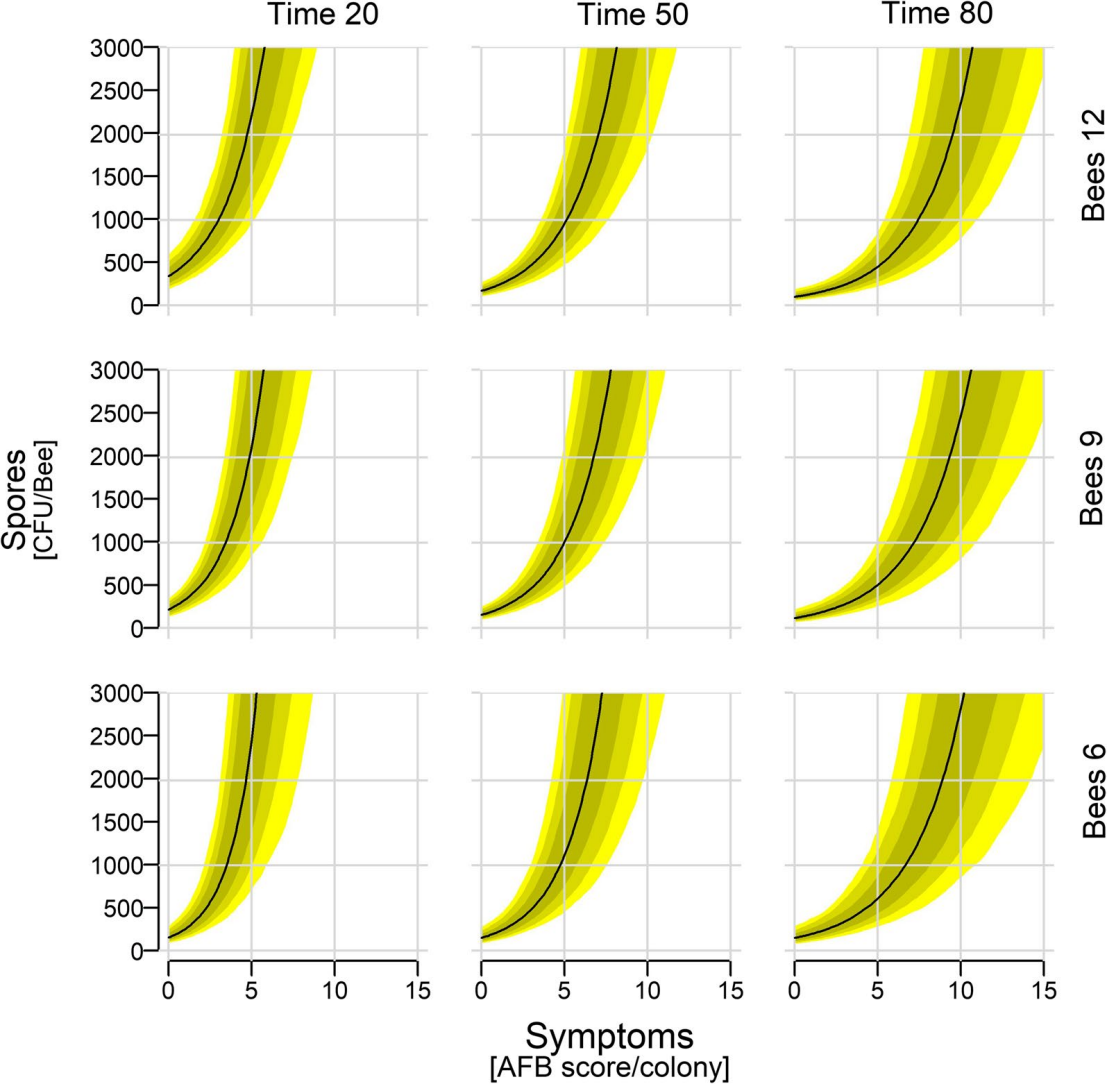
Spore counts depending on clinical symptoms, time of the season, and number of bees within the colony. Shown are median (with 97, 89, and 67% credible intervals) posterior distributions along the full range of observed AFB scores, but only until a maximum of 3000 Spores (see Additional file 2: Figure S4 for whole range of observed spore counts). The remaining continuous predictors are held approximately at their mean (Bees: 9.2; Time: 48.4), their 1st quantile (Bees: 6.0; Time: 21), and their 3rd quantile (Bees: 12.0; Time: 79)

Regardless of Time and Bees, Spores increased with increasing clinical symptoms (Fig. 2: Time 50/Bees 9; see also Additional file 2: Figure S4 for the full range of spore counts). In general though, Spores decreased over Time, as is illustrated by the decrease over Time for any given level of Symptoms (see also Additional file 2: Figure S5). Spores also decreased with increasing Bees irrespective of Time or Symptoms (Additional file 2: Figure S6). However, early in the season Spores increased with increasing number of bees.

Time was a stronger modulator of the Symptoms-Spores relationship than number of bees, since the model including only the Symptoms × Time interaction received 50% of the Akaike weight (Table 1). This is illustrated by a greater change in the slope of the Symptoms-Spores relationship in relation to Time than in relation to Bees (Fig. 2, Additional file 2: Figure S4).

We furthermore estimated the number of expected spores in an adult bee sample if symptoms are at level zero or level one, which resulted in around 158 and 228 spores, respectively. Lastly, we calculated the posterior of the difference for extreme and likely values of Spores in order to investigate a dilution effect of sampling 100 bees while colony size differed (see Additional file 2).

### Predicting the effect of AFB disease on colony strength

All response variables were relevant for predicting the first variable of colony size, the number of adult bees (Table 2). Brood was the strongest predictor of Bees, as revealed by the importance of its interactions with the other variables in all four selected models (Table 1). This importance is further illustrated by the high probability of an effect larger than zero after subtracting the effect of Spores or Time as a co-predictor. The time post-infection seemed to be a much more important co-predictor of the number of bees than the number of spores (Table 2).

**Fig. 3:**
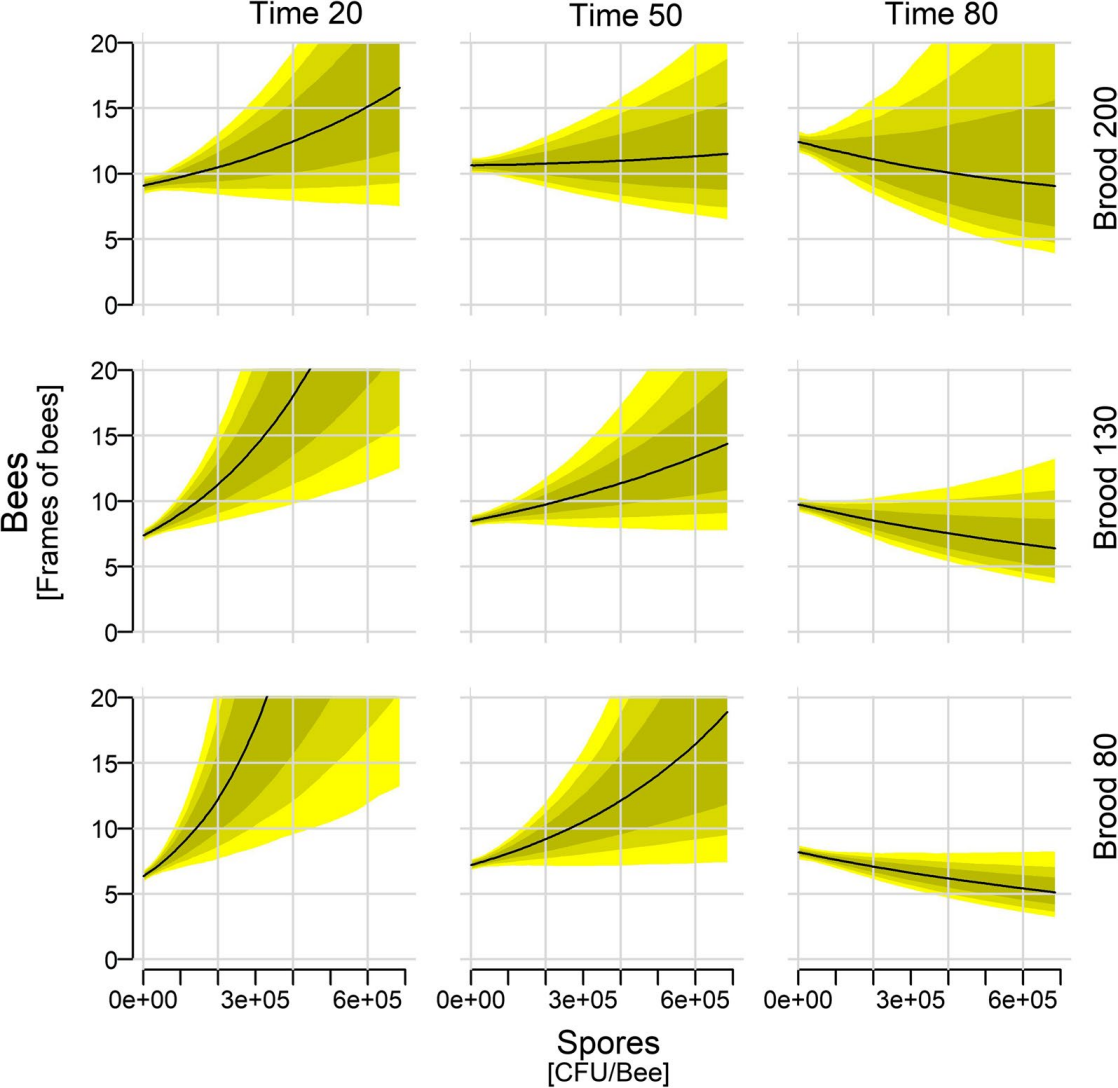
Colony size depending on spore count, time of the season, and brood size. Shown are median (with 97, 89, and 67% credible intervals) posterior distributions along the full range of observed spore counts. The remaining continuous predictors are held approximately at their mean (Brood: 132.6; Time: 48.4), their 1st quantile (Brood: 78; Time: 21), and their 3rd quantile (Brood: 191; Time: 79)

Regardless of Time or Brood the number of bees increased with increasing spore numbers (Fig. 3: Time 50/Brood 130). Similarly, regardless of Spores and Brood, Bees increased with Time (Fig. 3: see at zero spore count and in Additional file 2: Figure S7 at Spores 850) and Bees increase with increasing Brood if the other predictors are held at their mean/median values (Fig. 3: zero Time point; Additional file 2: Figure S8: Spores 850).

Time was a stronger modulator of the Bees-Spores relationship in the colonies than Brood as the model with the Spores × Time interaction was ranked higher than the model including the Spores × Brood interaction (Table 1). This is illustrated by the strong change over Time in the slope, from a positive to a negative relationship, while the changes with respect to Brood are less prominent (Fig. 3).

Regarding the second measure of colony strength, *i.e.* the amount of brood, we saw that all response variables were relevant for predicting brood amount (Table 2). The number of bees was by far the strongest predictor of brood amount in the first model, which included only the Symptoms × Bees interaction, and received 99% of all the predictors’ weight (Table 1). Therefore, here we only used M13 for the predictions instead of a weighted combination of all 4 models.

The importance of Bees for predicting the amount of brood is further illustrated by the high portability of an effect larger than zero after subtracting the co-predictor symptoms or time. Symptoms seemed more important than Time for predicting Brood (Table 2).

**Fig. 4:**
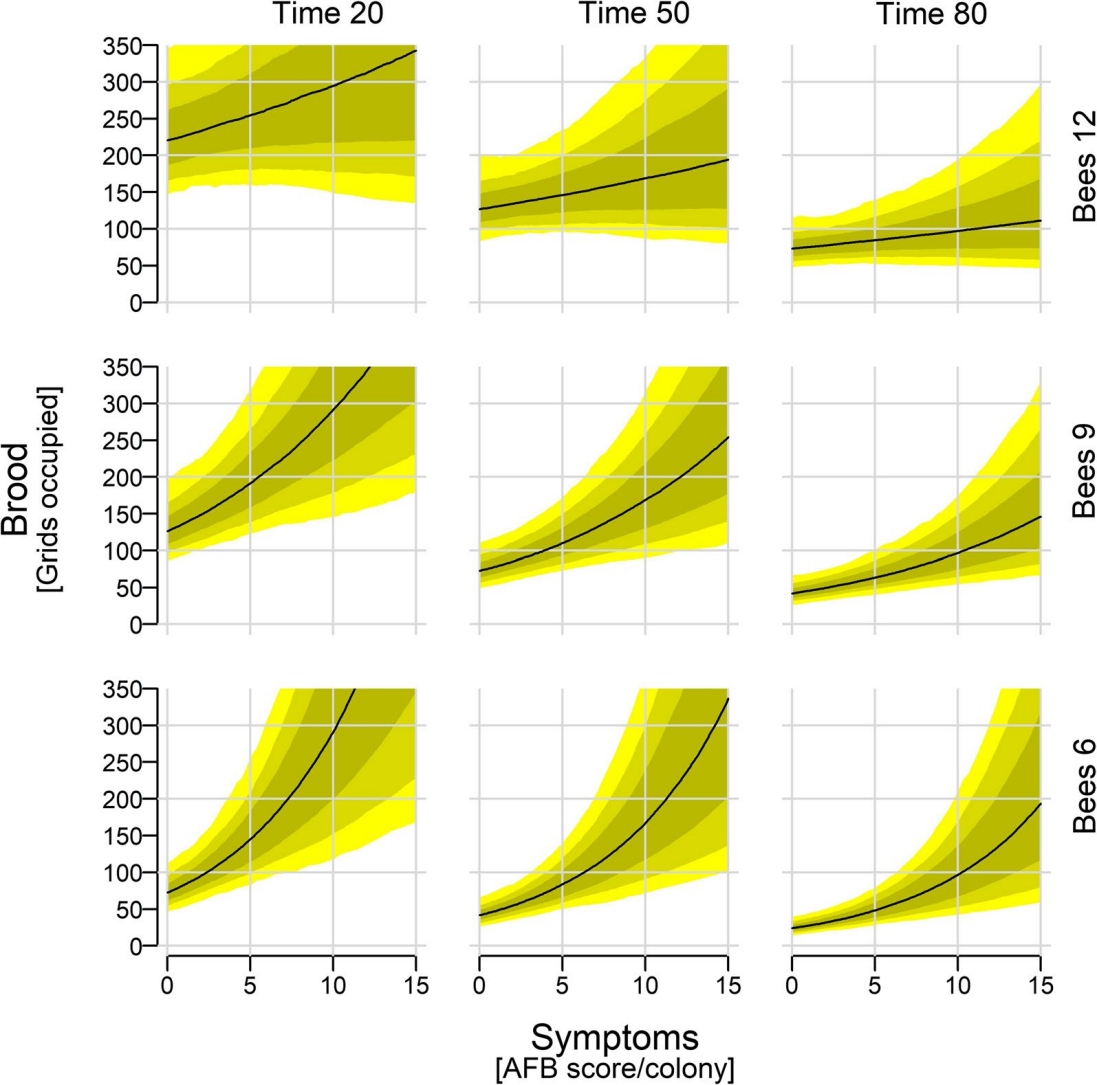
Brood size depending on spore count, time of the season, and colony size. Shown are median (with 97, 89, and 67% credible intervals) posterior distributions along the full range of observed clinical symptoms. The remaining continuous predictors are held approximately at their mean (Bees: 9.2; Time: 48.4), their 1st quantile (Bees: 6.0; Time: 21), and their 3rd quantile (Bees: 12.0; Time: 79)

Regardless of Time and Bees, the Brood increased with increasing Symptoms (Fig. 3: Time 50/Bees 9). Similarly, Brood decreased with Time irrespective of the Symptoms and Bees (Fig. 4 and Additional file 2: Figure S9, any symptom score). Brood also increases with Bees (Fig. 4), although at very high symptoms, more bees did not lead to more brood anymore.

Bees was an overwhelmingly stronger modulator of the Brood-Symptoms relationship relative to Time since Symptoms × Bees was the only relevant interaction (Table 1). This is illustrated by the strong change in the slope of the brood-symptoms relationship in relation to the number of Bees, relative to the slope in relation to Time (Fig. 4).

## Discussion

The primary objective of our study was to determine the probability distribution for encountering colony-level AFB symptoms from *P. larvae* spore counts, as a superior approach to identifying a threshold. By taking a probabilistic approach, other factors affecting the relationship between spore counts and symptoms, such as colony size, brood availability and stage of the infection process were explicitly accounted for, something which is not possible with simple threshold values.

While we could describe the relationship between spore levels and symptom independent of the effects of time of the season and the amount of brood (by keeping these at their mean value), questions remain about the generality of the results. Other possible factors that may change the spore-symptom relationship are variability in bacterial virulence (an innate genetic property of different *P. larvae* strains [54]), colony genetics [46], resistance against *P. larvae* [55], and hygienic behaviour [23]. All colonies in the study were placed in the same isolated apiary and were experimentally infected with sufficient *Paenibacillus larvae* spores to precipitate AFB disease. All colonies were therefore under the same infection pressure. This means that any additional contagion contributed by bees drifting between the colonies will be miniscule compared to the contagion developed within each colony, and will not have affected the results. Although proximity and connectedness (beekeeping and geolocation) are important determinants of the disease pressure in a colony [27] this is not applicable to the current study which was conducted in complete isolation from other beekeeping operations, as required by sanitary regulations.

From an applied perspective, it may be more interesting to get an idea of the spore counts given a symptom score. We found even for colony symptom scores of 0 (*i.e.* no disease), there is a 100% probability that the spore count is larger than zero, averaging around 158 Spores per bee (Table 2: *Symptoms *=* 0*). Symptomatic colonies with an AFB score of 1 corresponded to around 228 Spores per bee (Table 2: *Symptoms *=* 1*). This is considerably lower than the previously estimated threshold of 3000 spores per bee for AFB symptoms [37], but higher than the estimate of Lindström [36].

Scoring clinical symptoms may be biased given that symptoms may not be visible at an early stage of infection [56] and that symptoms in larger colonies with many brood frames to inspect may be underestimated by human eye [57, 58]. Regardless of the season or the spore levels, clinical symptoms increased with brood size in our study. It remains unclear if, and to what extent, the precision of the prediction of AFB symptoms suffers from increasing colony size. However, the AFB disease scoring is very sensitive at the lower range and any number of diseased cells above 100 corresponds the highest AFB symptom score, which would probably guard against such overestimation. Another result of our probabilistic approach is evidence that the AFB scoring method seems not to produce false positives, since for a spore count of 0, the probabilistic estimate for AFB symptoms does not reach 1, which is the minimum AFB-positive score (Table 2: *Spores *=* 0*: 0.22 ± 0.05). This confirms the accuracy of the scoring scale and previous findings that high spore levels will be detected in symptomatic colonies [28].

Our second objective was to clarify the interactive relationship between colony development and disease development. Brood amount and the time post infection were similarly important secondary predictive factors affecting the relationship between spore counts and symptoms. Both are important for the epidemiology of the disease: the amount of brood representing new, uninfected hosts and time being an obvious important factor in any epidemiological disease progression. Symptoms increased slowly with increasing brood size and over time. Including data from symptomatic colonies only would have shown a faster increase. However, we were also interested in predicting from the spore counts the probability of symptoms developing in colonies that passed visual AFB inspection. The presented models therefore investigated the disease development in infected colonies, rather than just in symptomatic colonies.

AFB kills progressively more brood as the epidemic intensifies, and the consequent shortage of new adult bees leads to progressive dwindling and eventual demise of the colony. This study describes this process in greater detail, including the behavioural responses of the colony. During normal colony development, and as long as there is forage available, more brood leads to more adult bees, which then leads to more brood and so on. In these experiments however, the amount of brood decreased throughout the entire season, rather than being restricted to the autumn (which is when the colonies normally transition to broodless winter colonies [59]). Admittedly we did not had uninfected control colonies the development of the brood could be compared to, which would have enabled us to estimate the brood loss over the season entirely attributed to AFB. Nevertheless, since no swarming occurred in these colonies during the season, we believe this decrease could therefore be (mostly) attributed to AFB. However, early in the season the colonies responded to AFB symptoms by increasing their brood production, shown by a greater increase in smaller colonies than in larger colonies (Fig. 4: steeper slope at Bees 6 than Bees 12). Nevertheless, later in the season this compensation attempt failed and the number of bees decreased with increasing bacterial spore levels (Fig. 3). This decrease may have been partly overestimated, due to the dilution effect in larger colonies, where the spore count per adult bee sample is shared between larger numbers of adult bees (see Additional file 2 for more explanations and implications for sampling strategy). The compensation failure can be seen more clearly by slower increase in brood amount with increasing symptoms severity (Fig. 4). Larger amounts of brood fail to hatch into adult workers, which is most clearly illustrated by the brood-to-bee ratio. At later time points, the same brood amount predicts a larger number of bees (Additional file 2: Figure S8) and the same number of bees predicts less brood (Additional file 2: Figure S10).

The observed increase in the amount of brood in response to a stressor has previously been described for *Varroa destructo*r infested colonies [14], where larger colonies in the autumn were more likely to die the following year. A bee colony is an adaptable unit that uses brood rearing as one of the mechanisms to respond to external and internal stimuli, through both positive and negative feedback loops [60]. A (perceived) deficit in either adults or healthy brood can be one of such internal stimuli, resulting in an elevated brood rearing effort in smaller colonies, and thus an elevated brood-to-adult ratio. We observed such a compensation attempt, at the most relevant time point of the life cycle for the colony development (spring), and we could show that this effect subsequently carried over to the adult (worker) bee stage. Although external factors such as foraging availability and quality, determined by the surrounding landscape, are also highly influential for brood rearing and overall colony strength [61, 62], all colonies in this study were located in the same apiary and therefore exposed to the same landscape and foraging conditions. Since the number of bees declined due to the disease we can also expect a feedback of lower food intake to further increase disease prevalence that creates a cycle of stress [63].

Our final objective was to investigate the epidemiological aspect of AFB. In these experiments we verify the expectation of the SIR model of epidemiology, but also identified peculiarities for the spread of AFB within a colony. The epidemic potential (given by the reproductive number R_0_) of a disease increases with increasing transmission rates and number of susceptible hosts [42]. The particularities of disease epidemiology in social animals was reviewed recently [45]. One theoretical prediction is that, contrary to the SIR model, disease prevalence may decrease with increasing group size, if the behavioural responses limiting disease prevalence or transmission become more effective with increasing group size, such as grooming behaviour in termites [64]. The hygienic behaviour of honeybees that involves detecting and removing infected and asymptomatic brood would also affect the SIR model [56], since it systematically reduces the amount of infectious material in the colony, while the disappearance of brood would of itself act as a stimulus for rearing new (uninfected) brood, both of which are important parameters for the SIR model. In these experiments, clinical symptoms always increased with brood size (Additional file 2: Figure S3), thus favouring the traditional SIR model of epidemiology as explanation. Although small colony size due to poor nutrition may amplify disease susceptibility [63], we do expect that the disease will be more severe and increase more over the season in larger colonies. This would mean larger colonies are not more resilient against AFB and will decrease in size stronger than smaller ones. Smaller colonies have lower expected overwintering survival [65] which could lead to an additional colony loss in the next spring.

Contrary to the positive effects of hygienic grooming behaviour on disease in termites, our study found a positive relationship between colony size and disease symptoms, implying the hygienic behaviour of brood removal is perhaps ineffective at breaking this relationship. In fact, the adults carry bacterial spores and serve as vectors infecting new brood. The broader implication here is whether group size facilitates or hinders disease transmission in social animals will depend on what life stage is affected by the disease and how this effect translates to the other life stages or affect the task allocation [66] within eusocial insects.

## Conclusions

We provide a novel, and potentially more reliable method for quantifying the relationship between *P. larvae* spore counts and AFB symptoms. Furthermore, we showed how AFB-caused brood mortality led to progressively fewer adult worker bees, eventually tipping the colony into a deadly negative spiral from which it could not escape. We identified that AFB disease epidemiology in honeybees follows the more traditional SIR model of epidemiology. We found little evidence of any beneficial effects of the hygienic behaviour of brood removal on containing the epidemic, especially since adult bees simultaneously also act as vectors of the disease. We extend the discussion further to larger eusocial societies exhibiting stronger social immunity by showing this seems not to apply for American foulbrood in honeybees as workers are removing diseased brood but also vectoring the disease. The study therefore emphasizes to consider how certain defence strategies will manifest themselves in other life stages of the eusocial society and shows the direct feedbacks between the epidemic over a season and the colony size.

## Methods

### Experimental design

On March 24th 2014, forty honeybee colonies located in an isolated apiary with a history of AFB in Beltsville, MD, USA were selected for the experiment (colonies owned by USDA-ARS Bee Research Lab). The experiment was originally designed to test the efficacy of a commercial honeybee specific lactic acid bacteria preparation against AFB relative to two negative controls (a placebo preparation and a no treatment control) and a positive control (the antibiotic Tylosin) (Additional file 3, [67]). The colonies were arranged in four rows of ten colonies each, with 1.5 m distance between individual colonies in each row and 1.5 m distance between rows and all entrances facing the same direction [68]. All colonies were experimentally inoculated with the same dose of *Paenibacillus larvae* spores at to precipitate AFB epidemics with the four treatment groups distributed randomly among the 40 colonies. Although no active measures were taken to prevent drifting between the 40 colonies, the uniform inoculation of all colonies in the apiary and the spatial randomization of the treatment groups means that the effect of drifting bees on the AFB development in colonies is both minimal, and randomly distributed between the treatment groups. The randomization also means that any potential variability in the data caused by the treatment groups can be accounted for statistically in our modelling (Additional file 2). The colonies were assessed and adult bees sampled on April 23rd, and then 21, 37, 51, 79, and 105 days after the first assessment. On each sampling occasion approximately 200 adult bees were collected from the brood chamber per colony and the samples were stored at − 20 ^°^C until spore estimation in the lab.

### Colony assessments, AFB scoring, and spore counting

On each of the six sampling occasions, total colony size, the amount of brood and the severity of AFB symptoms were evaluated using standard protocols. Colony size (hereafter: Bees; with capital first letter) was estimated by a cumulative score of the proportion of each frame side that was occupied by adult bees [69]. The amount of brood in the colony (hereafter: Brood) was estimated by a cumulative score of the number of 5 × 5 cm squares on each frame that were occupied by brood [70]. The colony-level severity of AFB (hereafter: Symptoms) was estimated by a cumulative score of the visual inspection of each brood frame for signs of the disease [38, 71]. Each frame was rated using the recommended scale of 0 (no visible signs), 1 (fewer than 10 diseased cells), 2 (11–100 diseased cells), and 3 (more than 100 diseased cells). Samples of diseased cells were tested in the laboratory to confirm the diagnoses. The spore levels (hereafter: Spores) were determined from samples of 100 adult worker bees, as described previously [32, 38]. The raw data consisted of *P. larvae* colony forming units (CFU) and the data are presented as CFU per bee (see also Additional file 3). The effect of colony or disease development during the season is represented in the models by Time.

### Data modelling

The data was obtained from 40 colonies, sampled once a month for 6 months during a single bee season (see Additional file 1 for original data overview). A Bayesian approach was used for the statistical modelling and analyses [72–74]. All variables were continuous counts and the analysis is similar to a multiple regression. The models were constructed in two steps (see Additional file 3: Model building and validation). First, two similar models with different random structures were compared. In step two, we compared eight models for each of the four response variables. Each model included the three main effects and all combinations of their interactions. The four most important models from step two were then used in the analysis by weighting the predictions in order to include modulations of one predictor by the other two predictors. To understand the effect of each predictor we calculated the posterior of the response variable along the full observed range of one explanatory variable while keeping the remaining two explanatory variables constant, conventionally at their mean/median value. For a better understanding of the complex interaction between the three continuous predictors two additional values were selected for each of the 4 models to investigate interactive effects. For example approximately the mean (132.6), 1st (78) and 3rd (191) quantile were used for brood (hereafter: Brood 130, Brood 80, Brood 200, respectively; see also Additional file 3: Model building and validation). Furthermore we selected specific values of a predictor and summarized the posterior of the response variable in order to answer specific questions (e.g.: Table 2: Symptoms at Spores level zero: *Spores *=* 0).* We also calculated three scenarios (*Extreme dilution*, *Likely dilution 1*, and *Likely dilution 2*) by subtracting the posterior of one set of values from the posterior of another set. The resulting posteriors can be seen as pairwise comparisons among these sets. In order to further investigate the predictiveness of each main effect compared to the other we subtracted the posteriors from each other [72]. The posterior of each main effect was weighted the same way as for the predictions and the smaller was subtracted from the larger in order to calculate the posterior of the difference. In all models we used minimal informative priors and the posterior was generated as a Monte Carlo sample (2000 iterations; Hamilton Monte Carlo; 1000 warm up, 1000 sampling the chains) using STAN [75] handled from R [76] using function from McElreath [73].

## Supplementary information

**Additional file 1:** Description of data used.

**Additional file 2:** Additional Figures S2 to S10.

**Additional file 3:** Colony treatment, spore counting, motivation of using Bayesian approach, model building and validation.

## Data Availability

The datasets generated during and/or analysed during the current study are available in the Zenodo repository, [https://zenodo.org/record/3672367] [77].

## References

[1] Gallai N, Salles JM, Settele J, Vaissière BE (2009). Economic valuation of the vulnerability of world agriculture confronted with pollinator decline. Ecol Econ.

[2] Hung K-LJ, Kingston JM, Albrecht M, Holway DA, Kohn JR (2018). The worldwide importance of honey bees as pollinators in natural habitats. Proc R Soc B Biol Sci.

[3] Aizen MA, Harder LD (2009). The global stock of domesticated honey bees is growing slower than agricultural demand for pollination. Curr Biol.

[4] Steinhauer N, Kulhanek K, Antúnez K, Human H, Chantawannakul P, Chauzat MP (2018). Drivers of colony losses. Curr Opin Insect Sci.

[5] McMenamin AJ, Genersch E (2015). Honey bee colony losses and associated viruses. Curr Opin Insect Sci.

[6] vanEngelsdorp D, Caron D, Hayes J, Underwood R, Henson M, Rennich K (2012). A national survey of managed honey bee 2010–2011 winter colony losses in the USA: results from the Bee Informed Partnership. J Apic Res.

[7] Gray A, Brodschneider R, Adjlane N, Ballis A, Brusbardis V, Charrière J-D (2019). Loss rates of honey bee colonies during winter 2017/2018 in 36 countries participating in the COLOSS survey, including effects of forage sources. J Apic Res.

[8] Tosi S, Nieh JC, Sgolastra F, Cabbri R, Medrzycki P (2017). Neonicotinoid pesticides and nutritional stress synergistically reduce survival in honey bees. Proc R Soc B Biol Sci.

[9] Doublet V, Labarussias M, de Miranda JR, Moritz RFA, Paxton RJ (2015). Bees under stress: sublethal doses of a neonicotinoid pesticide and pathogens interact to elevate honey bee mortality across the life cycle. Environ Microbiol.

[10] López JH, Krainer S, Engert A, Schuehly W, Riessberger-Gallé U, Crailsheim K (2017). Sublethal pesticide doses negatively affect survival and the cellular responses in American foulbrood-infected honeybee larvae. Sci Rep.

[11] Klein S, Cabirol A, Devaud J-M, Barron AB, Lihoreau M (2016). Why bees are so vulnerable to environmental stressors. Trends Ecol Evol.

[12] Becher MA, Osborne JL, Thorbek P, Kennedy PJ, Grimm V (2013). Towards a systems approach for understanding honeybee decline: a stocktaking and synthesis of existing models. J Appl Ecol.

[13] Vanengelsdorp D, Meixner MD (2010). A historical review of managed honey bee populations in Europe and the United States and the factors that may affect them. J Invertebr Pathol.

[14] van Dooremalen C, Cornelissen B, Poleij-Hok-Ahin C, Blacquière T (2018). Single and interactive effects of Varroa destructor, Nosema spp., and imidacloprid on honey bee colonies (*Apis mellifera*). Ecosphere.

[15] Straub L, Williams GR, Vidondo B, Khongphinitbunjong K, Retschnig G, Schneeberger A (2019). Neonicotinoids and ectoparasitic mites synergistically impact honeybees. Sci Rep.

[16] Genersch E, von der Ohe W, Kaatz H, Schroeder A, Otten C, Büchler R (2010). The German bee monitoring project: a long term study to understand periodically high winter losses of honey bee colonies. Apidologie.

[17] Chauzat MP, Jacques A, Laurent M, Bougeard S, Hendrikx P, EPILOBEE consortium (2016). Risk indicators affecting honeybee colony survival in Europe: one year of surveillance. Apidologie.

[18] Mill AC, Rushton SP, Shirley MDF, Smith GC, Mason P, Brown MA (2014). Clustering, persistence and control of a pollinator brood disease: epidemiology of American foulbrood. Environ Microbiol.

[19] Ebeling J, Knispel H, Hertlein G, Fünfhaus A, Genersch E (2016). Biology of *Paenibacillus larvae*, a deadly pathogen of honey bee larvae. Appl Microbiol Biotechnol.

[20] Genersch E (2010). American Foulbrood in honeybees and its causative agent, *Paenibacillus larvae*. J Invertebr Pathol.

[21] Hasemann L (1961). How long can spores of American foulbrood live?. Am Bee J.

[22] Lindström A, Korpela S, Fries I (2008). The distribution of *Paenibacillus larvae* spores in adult bees and honey and larval mortality, following the addition of American foulbrood diseased brood or spore-contaminated honey in honey bee (*Apis mellifera*) colonies. J Invertebr Pathol.

[23] Spivak M, Reuter GS (2001). Resistance to American foulbrood disease by honey bee colonies *Apis mellifera* bred for hygienic behavior. Apidologie.

[24] Wilson-Rich N, Spivak M, Fefferman NH, Starks PT (2009). Genetic, individual, and group facilitation of disease resistance in insect societies. Annu Rev Entomol.

[25] Bailey L, Ball B (1991). Honey bee pathology.

[26] Fries I, Camazine S (2001). Implications of horizontal and vertical pathogen transmission for honey bee epidemiology. Apidologie.

[27] Datta S, Bull JC, Budge GE, Keeling MJ (2013). Modelling the spread of American foulbrood in honeybees. J R Soc Interface.

[28] Gillard M, Charriere JD, Belloy L (2008). Distribution of *Paenibacillus larvae* spores inside honey bee colonies and its relevance for diagnosis. J Invertebr Pathol.

[29] Erban T, Ledvinka O, Kamler M, Nesvorna M, Hortova B, Tyl J (2017). Honeybee (*Apis mellifera*)-associated bacterial community affected by American foulbrood: detection of *Paenibacillus larvae* via microbiome analysis/631/158/855/631/326/2565/855/38/23/38/22/38/47 article. Sci Rep.

[30] Fries I, Lindström A, Korpela S (2006). Vertical transmission of American foulbrood (*Paenibacillus larvae*) in honey bees (*Apis mellifera*). Vet Microbiol.

[31] Lindström A, Fries I (2005). Sampling of adult bees for detection of American foulbrood (*Paenibacillus larvae* subsp larvae) spores in honey bee (*Apis mellifera*) colonies. J Apic Res.

[32] Forsgren E, Laugen AT (2014). Prognostic value of using bee and hive debris samples for the detection of American foulbrood disease in honey bee colonies. Apidologie.

[33] Nordström S, Forsgren E, Fries I (2002). Comparative diagnosis of American foulbrood using samples of adult honey bees and honey. Apic Sci.

[34] Goodwin RM, Perry JH, Haine HM (1996). A study on the presence of Bacillus larvae spores carried by adult honey bees to identify colonies with clinical symptoms of American foulbrood disease. J Apic Res.

[35] Del Hoyo ML, Basualdo M, Lorenzo A, Palacio MA, Rodriguez EM, Bedascarrasbure E (2001). Effect of shaking honey bee colonies affected by American foulbrood on *Paenibacillus larvae* larvae spore loads. J Apic Res.

[36] Lindström A (2008). Distribution of *Paenibacillus larvae* spores among adult honey bees (*Apis mellifera*) and the relationship with clinical symptoms of American foulbrood. Microb Ecol.

[37] Gende L, Satta A, Ligios V, Ruiu L, Buffa F, Fernandez N (2011). Searching for an American foulbrood early detection threshold by the determination of *paenibacillus larvae* spore load in worker honey bees. Bull Insectol.

[38] de Graaf DC, Alippi AM, Antúnez K, Aronstein KA, Budge G, De Koker D (2013). Standard methods for American foulbrood research. J Apic Res.

[39] Jatulan EO, Rabajante JF, Banaay CGB, Fajardo AC, Jose EC (2015). A mathematical model of intra-colony spread of American foulbrood in European honeybees (*Apis mellifera* L.). PLoS ONE.

[40] Pie MR, Rosengaus RB, Traniello JFA (2004). Nest architecture, activity pattern, worker density and the dynamics of disease transmission in social insects. J Theor Biol.

[41] Locke B, Fries I (2011). Characteristics of honey bee colonies (*Apis mellifera)* in Sweden surviving Varroa destructor infestation. Apidologie.

[42] Anderson RM, May RM (1982). Coevolution of hosts and parasites. Parasitology.

[43] Stephan JG, Low M, Stenberg JA, Björkman C (2016). Predator hunting mode and host plant quality shape attack-abatement patterns of predation risk in an insect herbivore. Ecosphere.

[44] Ezenwa VO, Worsley-Tonks KEL (2018). Social living simultaneously increases infection risk and decreases the cost of infection. Proc R Soc B Biol Sci.

[45] Schmid-Hempel P (2017). Parasites and their social hosts. Trends Parasitol.

[46] Tarpy DR (2003). Genetic diversity within honeybee colonies prevents severe infections and promotes colony growth. Proc R Soc B Biol Sci.

[47] Simone-Finstrom M, Walz M, Tarpy DR (2016). Genetic diversity confers colony-level benefits due to individual immunity. Biol Lett.

[48] Cremer S, Armitage SAO, Schmid-Hempel P (2007). Social Immunity. Curr Biol.

[49] Nunn CL, Jordan F, McCabe CM, Verdolin JL, Fewell JH (2015). Infectious disease and group size: more than just a numbers game. Philos Trans R Soc B Biol Sci.

[50] Tranter C, Lefevre L, Evison SEF, Hughes WOH (2015). Threat detection: contextual recognition and response to parasites by ants. Behav Ecol.

[51] Hoggard SJ, Wilson PD, Beattie AJ, Stow AJ (2013). The effectiveness of antimicrobial defenses declines with increasing group size and genetic similarity. Ann Entomol Soc Am.

[52] Donkersley P, Rhodes G, Pickup RW, Jones KC, Power EF, Wright GA (2017). Nutritional composition of honey bee food stores vary with floral composition. Oecologia.

[53] Walton A, Dolezal AG, Bakken MA, Toth AL (2018). Hungry for the queen: honey bee nutritional environment affects worker pheromone response in a life-stage dependent manner. Funct Ecol.

[54] Genersch E, Ashiralieva A, Fries I (2005). Strain- and genotype-specific differences in virulence of *Paenibacillus larvae* subsp. larvae, a bacterial pathogen causing American foulbrood disease in honeybees. Appl Environ Microbiol.

[55] Wedenig M, Riessberger-Gallé U, Crailsheim K (2003). A substance in honey bee larvae inhibits the growth of *Paenibacillus larvae* larvae. Apidologie.

[56] Brødsgaard CJ, Hansen H, Ritter W (2000). Progress of *Paenibacillus larvae* larvae infection in individually inoculated honey bee larvae reared singly in vitro, in micro colonies, or in full-size colonies. J Apic Res.

[57] Dakin SC, Tibber MS, Greenwood JA, Kingdom FAA, Morgan MJ (2011). A common visual metric for approximate number and density. Proc Natl Acad Sci.

[58] Jiménez J (2000). Effect of sample size, plot size, and counting time on estimates of avian diversity and abundance in a Chilean rainforest. J F Ornithol.

[59] Mattila HR, Otis GW (2007). Dwindling pollen resources trigger the transition to broodless populations of long-lived honeybees each autumn. Ecol Entomol.

[60] Winston ML (1987). The Biology of the honey bee.

[61] vanEngelsdorp D, Hayes J, Underwood RM, Pettis JS (2010). A survey of honey bee colony losses in the United States, fall 2008 to spring 2009. J Apic Res.

[62] Brodschneider R, Crailsheim K (2010). Nutrition and health in honey bees. Apidologie.

[63] Dolezal AG, Toth AL (2018). Feedbacks between nutrition and disease in honey bee health. Curr Opin Insect Sci.

[64] Rosengaus RB, Maxmen AB, Coates LE, Traniello JFA (1998). Disease resistance: a benefit of sociality in the dampwood termite *Zootermopsis angusticollis* (Isoptera: Termopsidae). Behav Ecol Sociobiol.

[65] Döke MA, McGrady CM, Otieno M, Grozinger CM, Frazier M (2019). Colony size, rather than geographic origin of stocks, predicts overwintering success in honey bees (Hymenoptera: Apidae) in the Northeastern United States. J Econ Entomol.

[66] Natsopoulou ME, McMahon DP, Paxton RJ (2016). Parasites modulate within-colony activity and accelerate the temporal polyethism schedule of a social insect, the honey bee. Behav Ecol Sociobiol.

[67] Stephan JG, Lamei S, Pettis JS, Riesbeck K, de Miranda JR, Forsgren E (2019). Honeybee-specific lactic acid bacterium supplements have no effect on American foulbrood-infected honeybee colonies. Appl Environ Microbiol.

[68] Dynes TL, Berry JA, Delaplane KS, Brosi BJ, De Roode JC (2019). Reduced density and visually complex apiaries reduce parasite load and promote honey production and overwintering survival in honey bees. PLoS ONE.

[69] Delaplane KS, van der Steen J, Guzman-Novoa E (2013). Standard methods for estimating strength parameters of *Apis mellifera* colonies. J Apic Res.

[70] Pettis JS, Rose R, Chaimanee V (2017). Chemical and cultural control of *Tropilaelaps mercedesae* mites in honeybee (*Apis mellifera*) colonies in Northern Thailand. PLoS ONE.

[71] Pettis JS, Feldlaufer MF (2005). Efficacy of lincomycin and tylosin in controlling American foulbrood in honey bee colonies. J Apic Res.

[72] Kruschke JK, Aguinis H, Joo H (2012). The time has come: Bayesian methods for data analysis in the organizational sciences. Organ Res Methods.

[73] McElreath R (2015). Statistical rethinking: a Bayesian course with examples in R and Stan. J Educ Behav Stat.

[74] Kruschke JK (2014). Doing Bayesian data analysis: A tutorial with R, JAGS, and Stan.

[75] Stan Development Team. Stan Modeling Language Users Guide and Reference Manual, Version 2.17.0. 2017. http://mc-stan.org/.

[76] R Core Team. R: A language and environment for statistical computing. 2017. https://www.r-project.org.

[77] Stephan JG, de Miranda JR, Forsgren E (2020). Data_Rcode for: American foulbrood in a honeybee colony: spore-symptom relationship and feedbacks between disease and colony development. BMC Ecology.

