# Correction to: American foulbrood in a honeybee colony: spore-symptom relationship and feedbacks between disease and colony development

**DOI:** 10.1186/s12898-020-00285-8

**Published:** 2020-03-23

**Authors:** Jörg G. Stephan, Joachim R. de Miranda, Eva Forsgren

**Affiliations:** 1 Department of Ecology, Swedish University of Agricultural Sciences, 750 07 Uppsala, Sweden; 2 Swedish Species Information Centre, Swedish University of Agricultural Sciences, 750 07 Uppsala, Sweden

## Correction to: BMC Ecol (2020) 20:15 10.1186/s12898-020-00283-w

Unfortunately, the original version of the article [1] contained an error. The author has brought to our attention that the article title is truncated in the published version. The correct title is *American foulbrood in a honeybee colony: spore*-*symptom relationship and feedbacks between disease and colony development.* Instead, it was published inadvertently as *American foulbrood in a honeybee colony: spore symptom relationship and feedbacks* due to an error occurred during the production process.

The original article has been corrected.
