# Supplementary material for: American foulbrood in a honeybee colony: spore-symptom relationship and feedbacks
Source: BMC Ecol. 2020 Mar 6;20:15. doi: 10.1186/s12898-020-00283-w (PMC7060557; doi:10.1186/s12898-020-00283-w)
Supplement: Supplementary file 1 — Additional file 1: Description of data used. [file 12898_2020_283_MOESM1_ESM.docx]

**Additional file 1**

**Description of data used**

**Title:**

American foulbrood in a honeybee colony: spore-symptom relationship and feedbacks between disease and colony development

Jörg G Stephan^a,b,*^, Joachim R. de Miranda^a^, Eva Forsgren^a^

^a^ Department of Ecology, Swedish University of Agricultural Sciences, Uppsala, 750 07 Sweden

^b^ Swedish Species Information Centre, Swedish University of Agricultural Sciences, Uppsala, 750 07 Sweden

* Corresponding author: jorg.stephan@slu.se


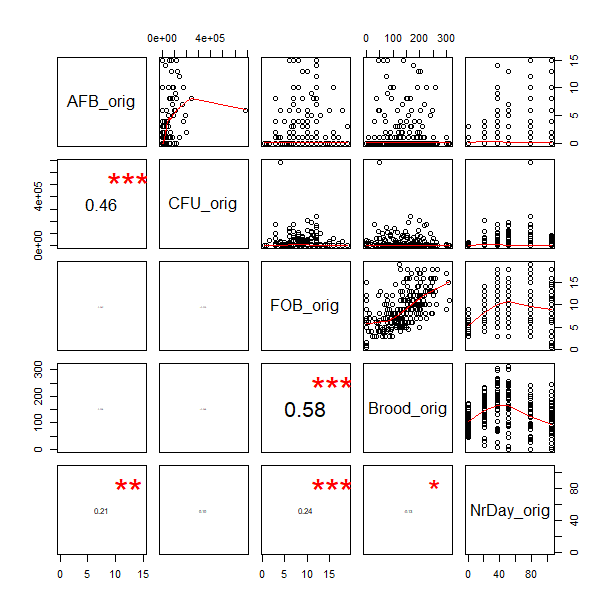


Figure S1: Original data of the five variables plotted against each other on the original scale. Red lines indicate Lowess smoother and numbers show Pearson correlation coefficient and significance (* p<0.05; ** p<0.01; *** p<0.001).

Table S1: Summary statistic of original data.

|  | Treatment | n | mean | sd | median | min | max | se |
| --- | --- | --- | --- | --- | --- | --- | --- | --- |
| AFB | Control | 60 | 2.4 | 4.2 | 0 | 0 | 15 | 0.55 |
|  | LAB | 60 | 1.5 | 3.3 | 0 | 0 | 15 | 0.42 |
|  | LABc | 58 | 1.9 | 3.4 | 0 | 0 | 13 | 0.45 |
|  | Tylosin | 59 | 0.53 | 1.2 | 0 | 0 | 6 | 0.16 |
| CFU | Control | 60 | 21000 | 40000 | 917 | 0 | 202667 | 5100 |
|  | LAB | 60 | 25000 | 45000 | 872 | 0 | 191000 | 5800 |
|  | LABc | 58 | 35000 | 100000 | 600.5 | 0 | 685000 | 13000 |
|  | Tylosin | 59 | 8300 | 21000 | 1000 | 0 | 139000 | 2700 |
| Brood | Control | 60 | 140 | 65 | 138 | 0 | 267 | 8.3 |
|  | LAB | 60 | 130 | 84 | 141 | 0 | 307 | 11 |
|  | LABc | 58 | 130 | 75 | 125.5 | 0 | 299 | 9.8 |
|  | Tylosin | 59 | 130 | 74 | 128 | 0 | 312 | 9.6 |
| NrDay | Control | 60 | 49 | 35 | 44 | 1 | 105 | 4.5 |
|  | LAB | 60 | 49 | 35 | 44 | 1 | 105 | 4.5 |
|  | LABc | 58 | 48 | 35 | 37 | 1 | 105 | 4.6 |
|  | Tylosin | 59 | 48 | 35 | 37 | 1 | 105 | 4.5 |
| FOB | Control | 60 | 9.5 | 3.3 | 9 | 3 | 18 | 0.43 |
|  | LAB | 60 | 9.7 | 5 | 10 | 1 | 19 | 0.64 |
|  | LABc | 58 | 8.9 | 4.1 | 8 | 3 | 19 | 0.54 |
|  | Tylosin | 59 | 8.5 | 3.6 | 8 | 0.5 | 18 | 0.46 |
