# Supplementary material for: American foulbrood in a honeybee colony: spore-symptom relationship and feedbacks
Source: BMC Ecol. 2020 Mar 6;20:15. doi: 10.1186/s12898-020-00283-w (PMC7060557; doi:10.1186/s12898-020-00283-w)
Supplement: Supplementary file 2 — Additional file 2: Additional Figures S2 to S10. [file 12898_2020_283_MOESM2_ESM.docx]

**Additional file 2**

**Supplemental figures S2 to S10**

**Title:**

American foulbrood in a honeybee colony: spore-symptom relationship and feedbacks between disease and colony development

Jörg G Stephan^a,b,*^, Joachim R. de Miranda^a^, Eva Forsgren^a^

^a^ Department of Ecology, Swedish University of Agricultural Sciences, Uppsala, 750 07 Sweden

^b^ Swedish Species Information Centre, Swedish University of Agricultural Sciences, Uppsala, 750 07 Sweden

* Corresponding author: jorg.stephan@slu.se


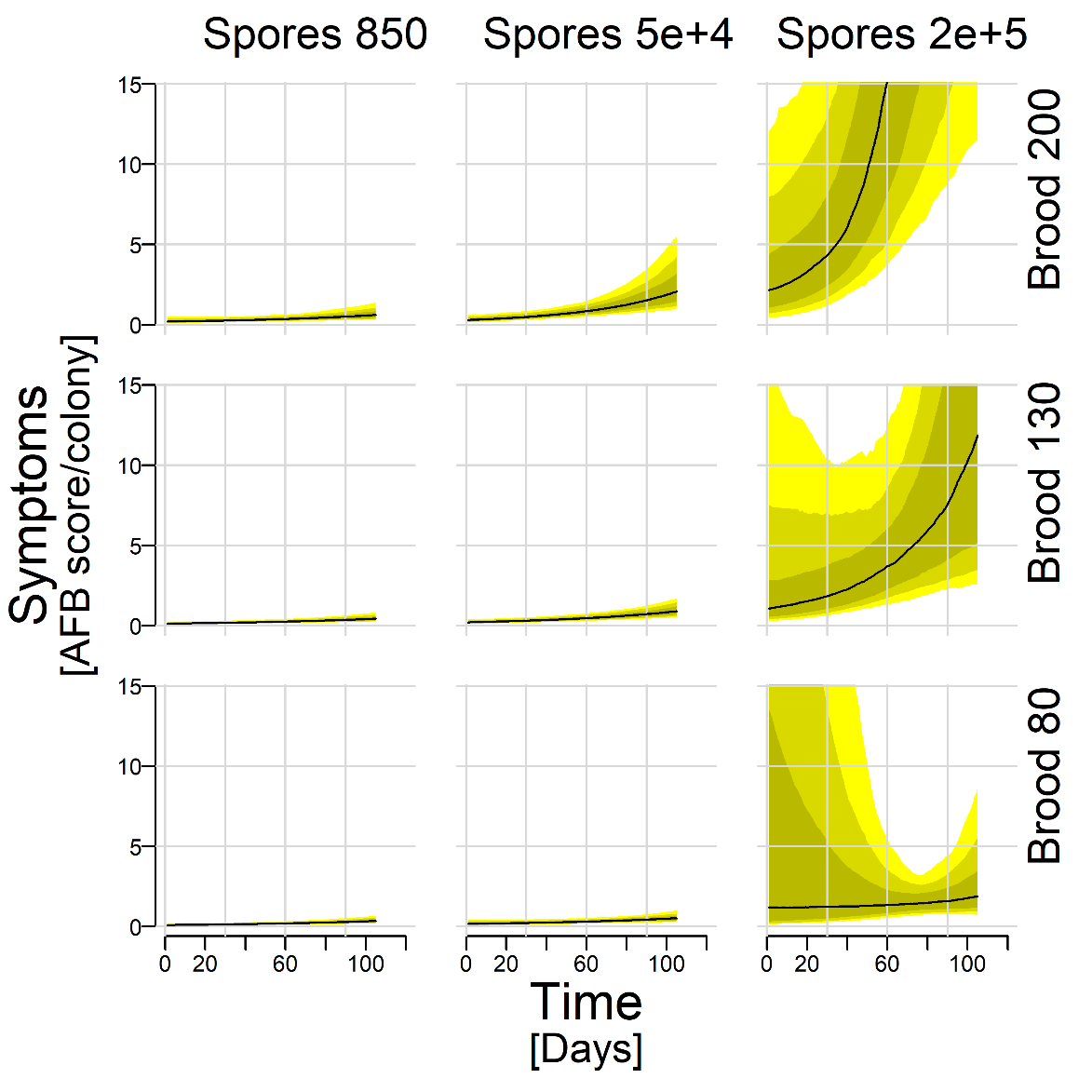


Fig. S2: Clinical symptoms depending on time of the season. Shown are median (with 97, 89, and 67 % credible intervals) posterior distributions along the full range of observation time. Brood sizes are held approximately at their mean (132.6), their 1^st^ quantile (78), and 3^rd^ quantile (191). Spore counts are held approximately at their median (834) and the values 50000 and 200000. Predictions are weighted predictions form four models with different combinations of the three explanatory variables and their interactions.

**
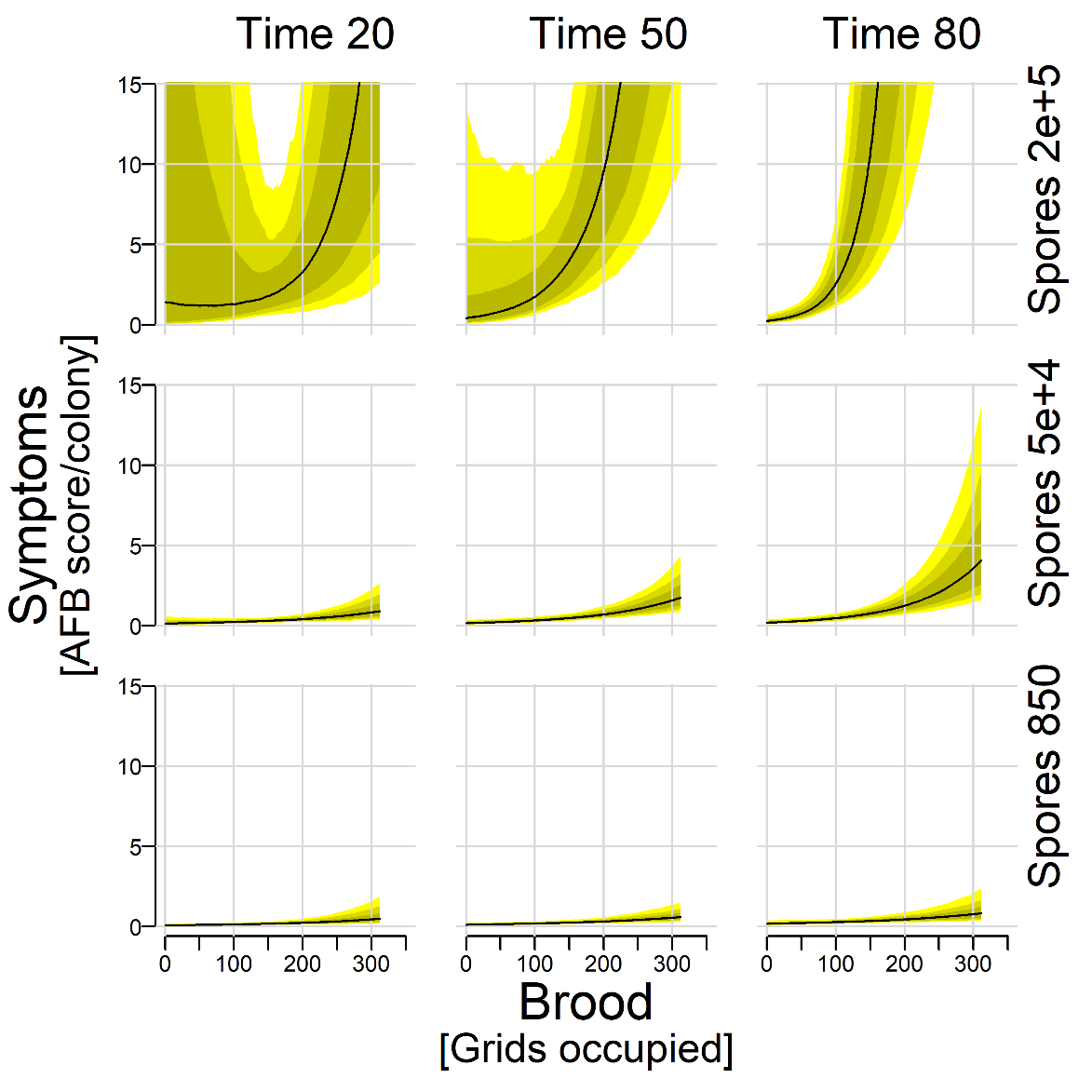
**

Fig. S3: Clinical symptoms depending on brood size. Shown are median (with 97, 89, and 67 % credible intervals) posterior distributions along the full range of brood size. Days are held approximately at their mean (48.4), their 1^st^ quantile (21), and 3^rd^ quantile (79). Spore counts are held approximately at their median (834) and the values 50000 and 200000. Predictions are weighted predictions form four models with different combinations of the three explanatory variables and their interactions.


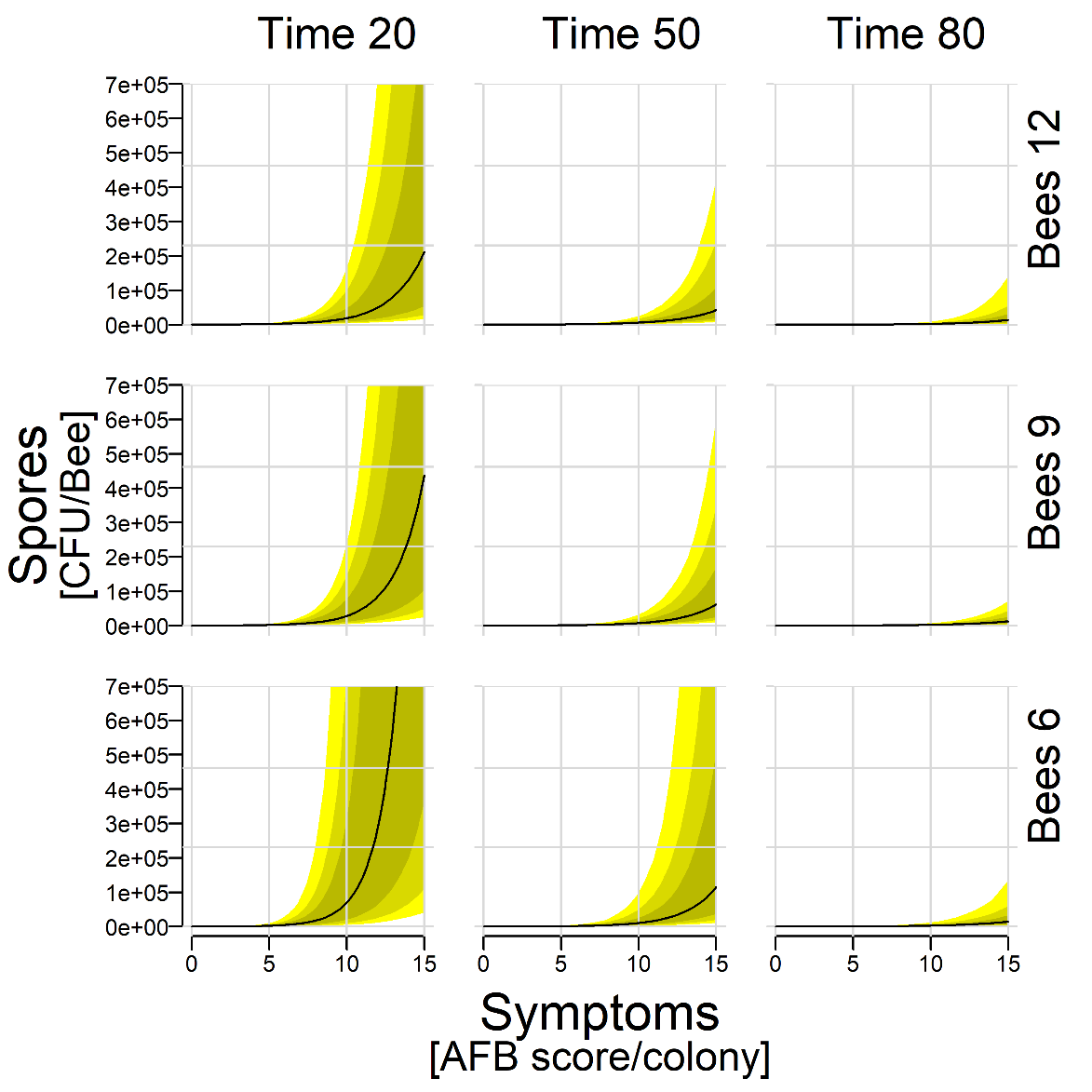


Fig. S4: Spore counts depending on clinical symptoms, time of the season, and number of bees within the colony (FOB = frames of bees). Shown are median (with 97, 89, and 67 % credible intervals) posterior distributions along the full range of observed AFB scores. The remaining continuous predictors are held approximately at their mean (Bees: 9.2; Time: 48.4), their 1^st^ quantile (Bees: 6.0; Time: 21), and their 3^rd^ quantile (Bees: 12.0; Time: 79). Predictions are weighted predictions form four models with different combinations of the three explanatory variables and their interactions.

**
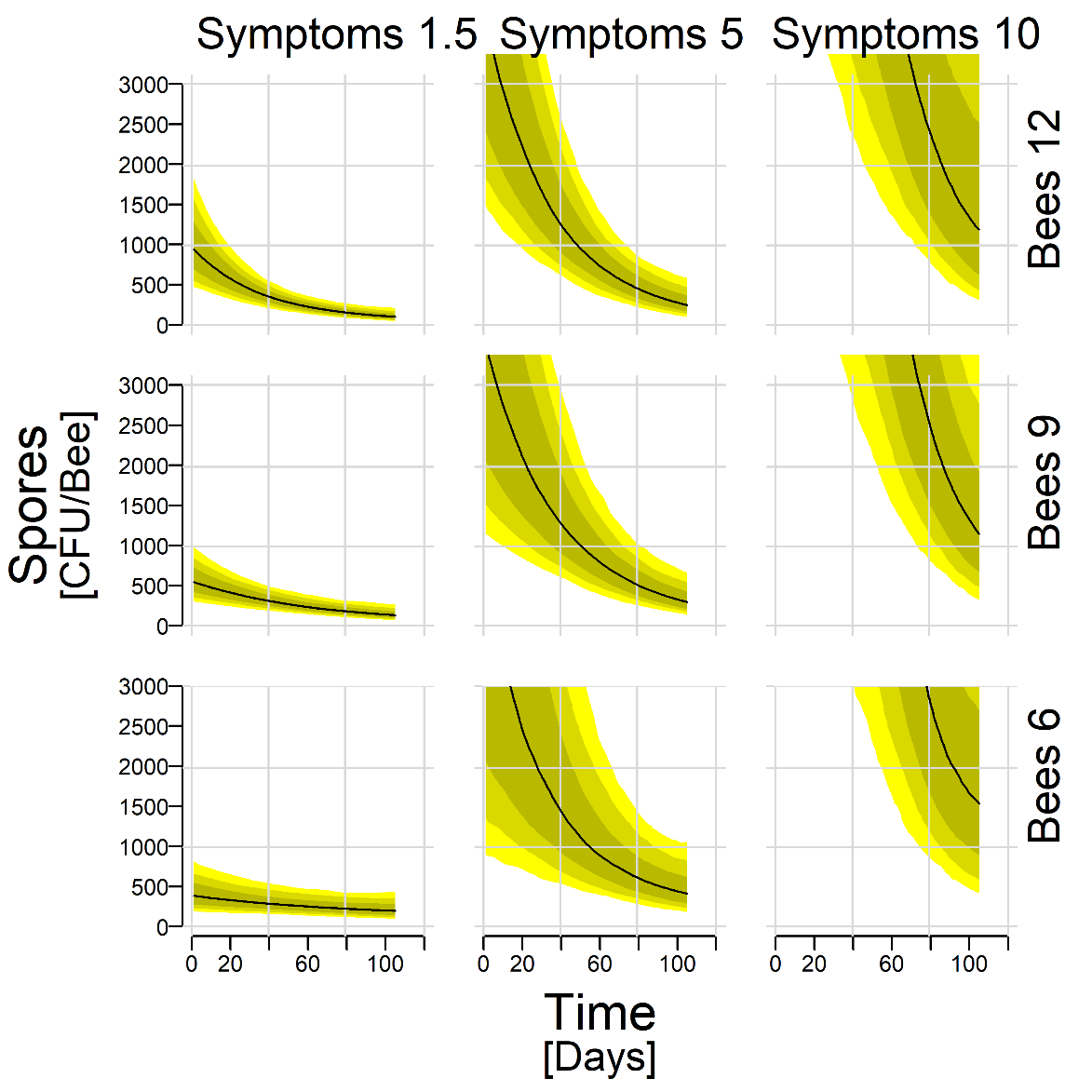
**

Fig. S5: Spore counts depending on clinical symptoms, time of the season of colony, and number of bees within the colony. Shown are median (with 97, 89, and 67 % credible intervals) posterior distributions along the time of the season. Number of bees are held approximately at their mean (Bees: 9.2), their 1^st^ quantile (Bees: 6.0), and their 3^rd^ quantile (Bees: 12.0). Symptoms are held approximately at their median (1.5) and the values 5 and 10. Predictions are weighted predictions form four models with different combinations of the three explanatory variables and their interactions.

**
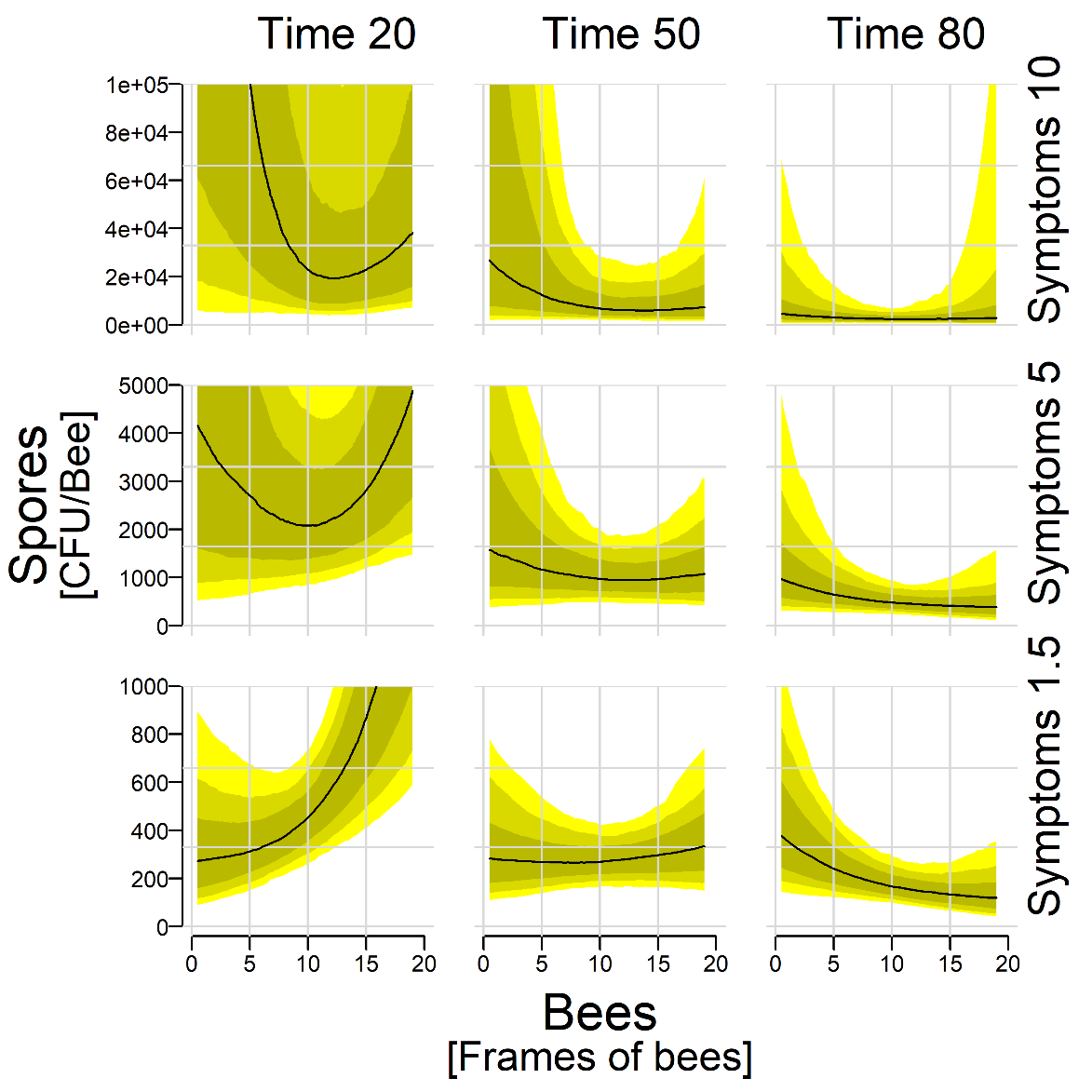
**

Fig. S6: Spore counts depending on clinical symptoms, time of the season, and number of bees within the colony. Shown are median (with 97, 89, and 67 % credible intervals) posterior distributions along the full range of observed number of bees. Time is held approximately at its mean (48.4), their 1^st^ quantile (21), and 3^rd^ quantile (79). Symptoms are held approximately at their median (1.5) and the values 5 and 10. Predictions are weighted predictions form four models with different combinations of the three explanatory variables and their interactions. Mind that ranges of y axis are chosen in order to see the relationship.


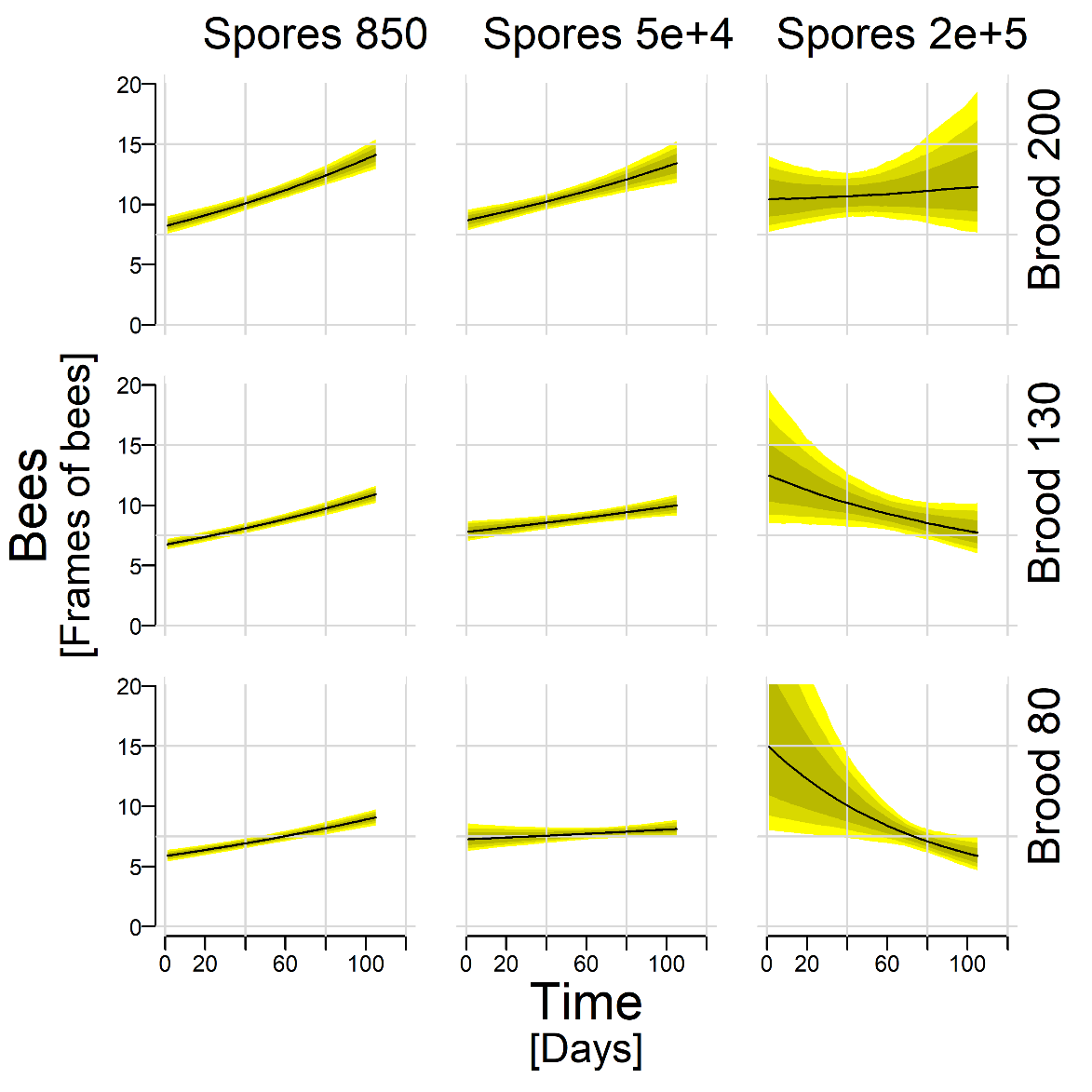


Fig. S7: Colony size depending on spore count, time of the season of colony, and brood size. Shown are median (with 97, 89, and 67 % credible intervals) posterior distributions along the time of the season. Brood sizes are held approximately at their mean (132.6), their 1st quantile (78), and 3rd quantile (191). Spore counts are held approximately at their median (834) and the values 50000 and 200000. Predictions are weighted predictions form four models with different combinations of the three explanatory variables and their interactions.

**
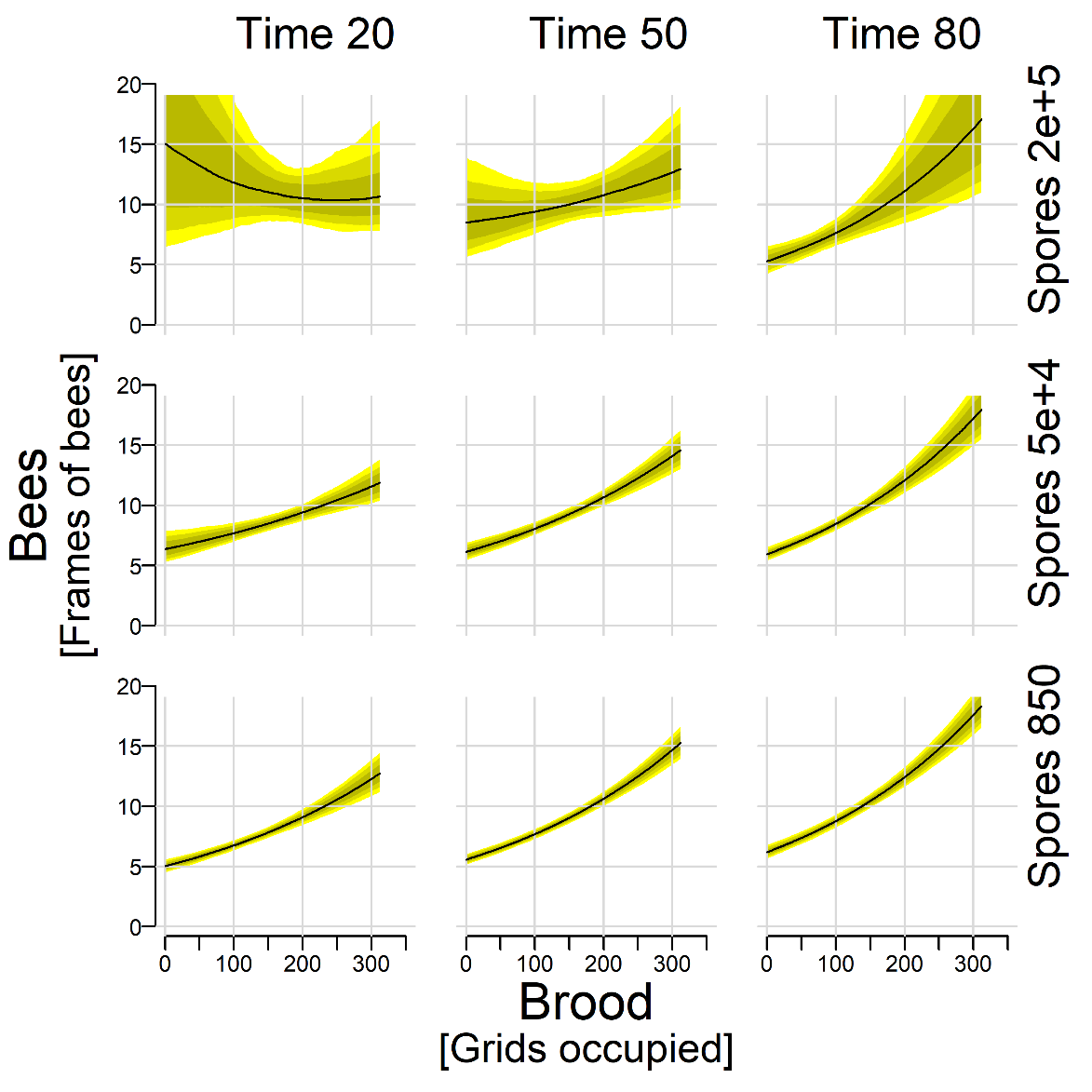
**

Fig. S8: Colony size depending on spore count, time of the season of colony, and brood size. Shown are median (with 97, 89, and 67 % credible intervals) posterior distributions along the full range of observed brood size. Time is held approximately at its mean (48.4), their 1^st^ quantile (21), and 3^rd^ quantile (79). Spore counts are held approximately at their median (834) and the values 50000 and 200000. Predictions are weighted predictions form four models with different combinations of the three explanatory variables and their interactions.


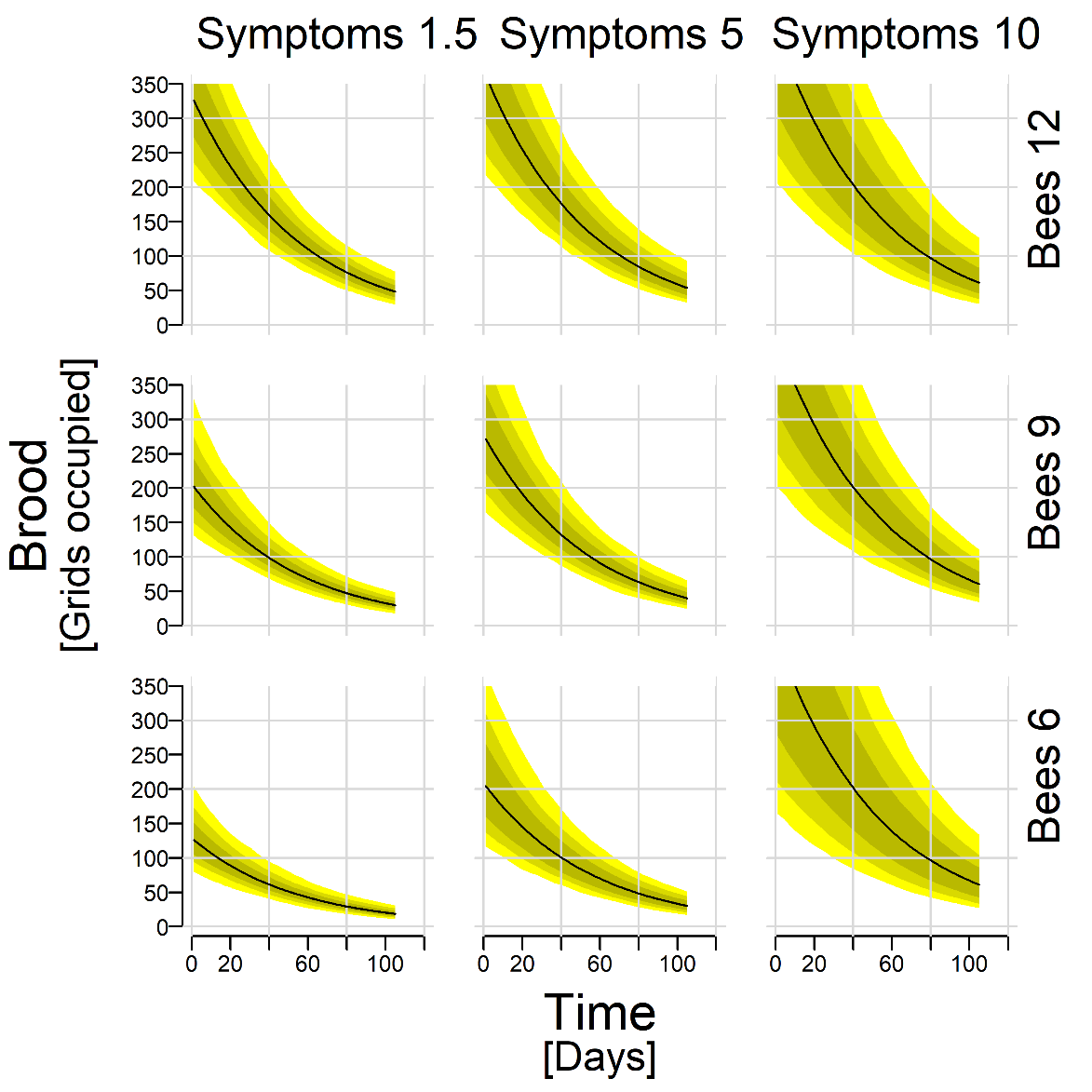


Fig. S9: Brood size depending on spore count, time of the season of colony, and colony size. Shown are median (with 97, 89, and 67 % credible intervals) posterior distributions along the full range of observed colony size. Number of bees are held approximately at their mean (Bees: 9.2), their 1^st^ quantile (Bees: 6.0), and their 3^rd^ quantile (Bees: 12.0). Symptoms are held approximately at their median (1.5) and the values 5 and 10. Predictions are weighted predictions form four models with different combinations of the three explanatory variables and their interactions.


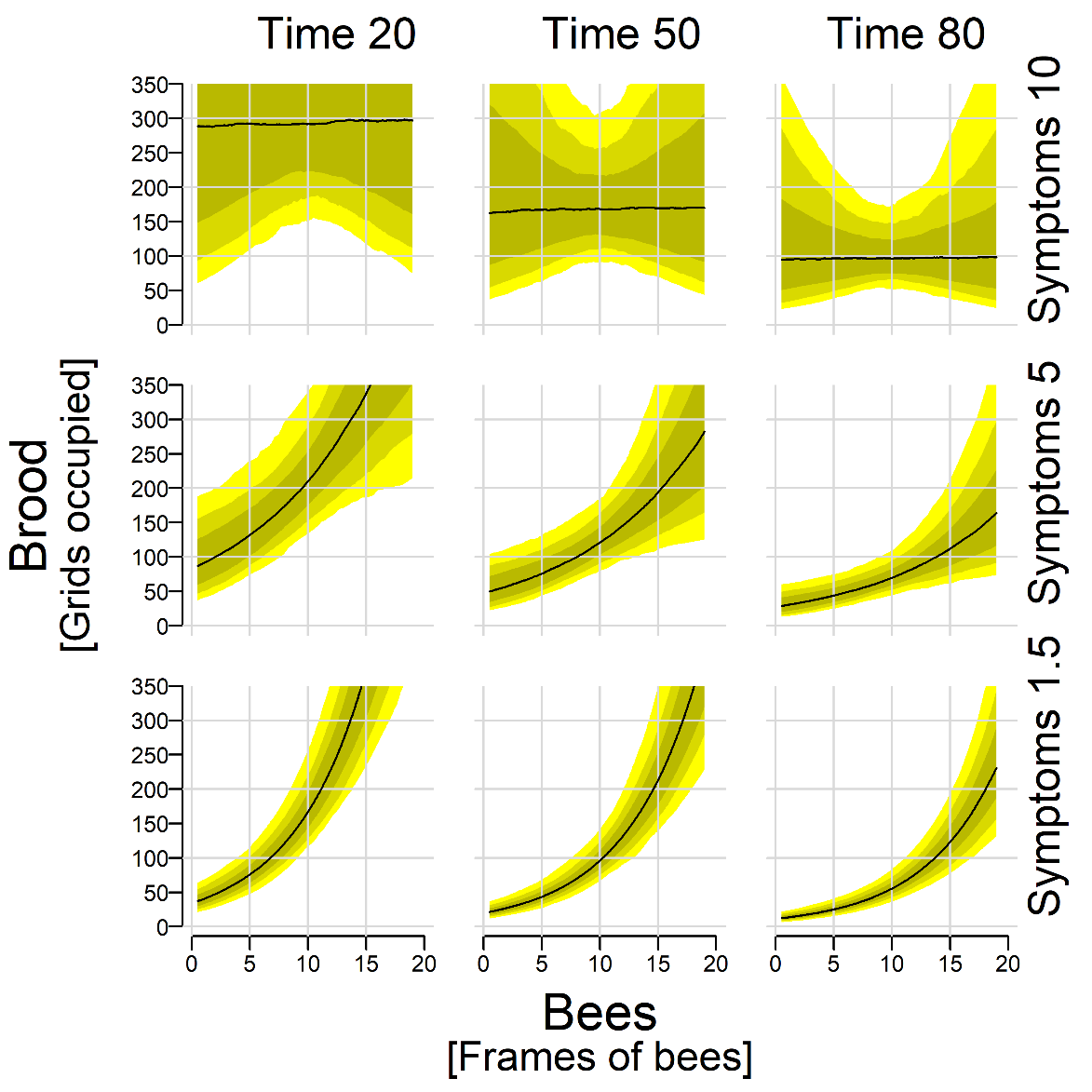


Fig. S10: Brood size depending on spore count, time of the season of colony, and colony size. Shown are median (with 97, 89, and 67 % credible intervals) posterior distributions along the time of the season. Time is held approximately at its mean (48.4), their 1^st^ quantile (21), and 3^rd^ quantile (79). Symptoms are held approximately at their median (1.5) and the values 5 and 10. Predictions are weighted predictions form four models with different combinations of the three explanatory variables and their interactions.
