# Supplementary material for: American foulbrood in a honeybee colony: spore-symptom relationship and feedbacks
Source: BMC Ecol. 2020 Mar 6;20:15. doi: 10.1186/s12898-020-00283-w (PMC7060557; doi:10.1186/s12898-020-00283-w)
Supplement: Supplementary file 3 — Additional file 3: Colony treatment, spore counting, motivation of using Bayesian approach, model building and validation. [file 12898_2020_283_MOESM3_ESM.docx]

**Additional file 3**

**Colony treatment, Spore counting, Motivation of using Bayesian approach, Model building and validation**

**Title:**

American foulbrood in a honeybee colony: spore-symptom relationship and feedbacks between disease and colony development

Jörg G Stephan^a,b,*^, Joachim R. de Miranda^a^, Eva Forsgren^a^

^a^ Department of Ecology, Swedish University of Agricultural Sciences, Uppsala, 750 07 Sweden

^b^ Swedish Species Information Centre, Swedish University of Agricultural Sciences, Uppsala, 750 07 Sweden

* Corresponding author: jorg.stephan@slu.se

*Colony treatment*

The colonies were assessed and adult bees sampled on April 23^rd^, and then 21, 37, 51, 79, and 105 days after the first assessment. On each sampling occasion approximately 200 adult bees were collected from the brood chamber per colony and the samples were stored at -20^°^C until spore estimation in the lab.

The experiment was originally designed to test the effect of the antibiotic tylosin and honey specific lactic acid bacteria on AFB [1]. All colonies received the same amount of food in the previous autumn and were fed three times with a 1:1 sucrose:water solution meaning the starting conditions of the colonies were comparable. The colonies were divided into four groups of ten colonies each (tylosin treatment, hbsLAB treatment, hbsLAB placebo and untreated control) using a stratified random design. On April 23 the colonies were inspected for the presence of AFB and on May 1^st^ all colonies were inoculated with a suspension of *P. larvae* spores to boost the onset of AFB. This was done by spraying two combs of unsealed brood with 5 ml of a sucrose solution containing the spores (approximately 200 million *P. larvae* spores per colony). The hbs-LAB treatment/placebo was administered twice, on April 23-25 and May 7-9, while the Tylosin treatment was administered on May 13^th^. The effects of the various treatments on AFB and colony development have been presented in a separate publication [1]. Although some treatments affected some of the predictors (e.g.: the treatment tylosin lowered the clinical symptoms over all sampling occasions [1]) we decided to include all colonies rather than excluding a fourth of all data for several reasons. First we modelled the variability originating from the treatments (see below) and, secondly, were interested in modulations of the relationship between two predictors by a third predictor. Excluding one treatment would mean excluding the data from all other predictors that are unaffected by the treatment meaning the interactive effects would have been less precise. Lastly, if symptoms are slightly higher while spore counts are not affected would mean we slightly overestimated the spore count at symptom levels of zero and the minimum detection. For example the current model resulted in 228 spores at the minimal AFB score of 1, which was already considerable lower than previous studies and a lower count of e.g. 100 spores is irrelevant given the magnitudes of spore counts.

*Spore counting*

Samples of 100 adult worker bees were crushed in 20 mL of sterile 0.9 % NaCl in a filter grinding bag (Neoreba®). The fluid produced was centrifuged for 10 min at 27,000g and the resulting pellet was re-suspended in 2 mL sterile NaCl, heat shocked at 85 °C for 10 min and spread out over 3 MYPGP-agar plates (10 µL each). The numbers of *P. larvae* colonies were counted on each plate after an incubation period of 7 days at 36 °C in 5 % CO_2_ and a mean value for the 3 plates calculated. The numbers of *P. larvae* bacteria colony forming units (CFU) were counted and the data presented as CFU per bee.

Motivation of using Bayesian approach

A Bayesian approach was used for the statistical modelling and analyses, since this examines the validity (probability) of a hypothesis (*i.e.* “spore levels can predict symptoms”) given the available data, as opposed to the frequentist approach (and P-value use), which examines the validity of the data for rejecting a null hypothesis [2–4]. Generally speaking, the Bayesian approach produces more reliable and accurate predictions and the results are posterior probability distributions from which probabilistic statements about the size and direction of an effect can be made.

Model building and validation

We used a large data set recording the development of AFB disease in experimentally infected colonies across a single bee season, as well as the effects of the disease on colony development, in order to evaluate the ability of certain variables to predict the outcome of the other variables.

The data involved 237 observations (40 colonies * 6 sampling occasions minus 3 missing observations due to colony death). Thirteen of these observations were free of *P. larvae* spores. However, since these observations were randomly distributed in time, between colonies and between treatment groups, all colonies were considered to be infected for the statistical analyses.

When used as response the variables were modelled with a Poisson likelihood (log link function). Although Symptoms represents a mixture of ordinal (0-3) and continuous (sum of 0-3 per frame for each colony) scales, the latter was used here since it more accurately represents the total AFB burden in the colony and is therefore the logical counterpart to the colony-level spore count. Hence, all predictor variables were used as continuous variables and were scaled and centred [5].

The models were constructed in two steps. First, two similar models with different random structures were compared, where the first model included just colony ID and every data point, while the second model also included the treatment groups, as Gaussian random effects. Each observation was assigned its own likelihood which removed the possibility of model over-dispersion [6] while the inclusion of colony ID accounted for the repeated measure structure across time. The difference between the two models’ predictions identifies the extent to which the different treatment groups affected our conclusions. These comparisons showed that only in the models for Symptoms was it important to specifically account for the different treatment groups. This is logical, since one of the treatments, tylosin, affects AFB symptoms, but not spore levels [1]. For the other models, colony ID and the individual observations sufficed as random effects. In step two, we compared eight models for each of the four response variables. Each model included the three main effects (Time, a predictor of colony strength and the most relevant predictor for AFB prevalence) and all combinations of their interactions. For example the model on Brood used Symptoms as most relevant disease predictor, since clinical symptoms only occur in the brood. The four most important models were then used in the analysis by weighting the predictions in order to include modulations of one predictor by the other two predictors. The weighted predictions of these four models were subsequently back-transformed to the original scale. To understand the effect of each predictor we calculated the posterior of the response variable along the full observed range of one explanatory variable while keeping the remaining two explanatory variables constant, conventionally at their mean/median value. For a better understanding of the complex interaction between the three continuous predictors two additional values were selected for each of the 4 models to investigate interactive effects. The selection of all tree values was straight-forward for Brood, Bees, and Time (mean, 1^st^ and 3^rd^ quantile), but less so for the highly skewed distributions of Spores and Symptoms. The mean (1.5), 5, and 10 AFB scores were used for Symptoms, while the median (~850), 50000, and 200000 spores per bee were used for Spores. Nevertheless, each variable was also used on the x axis but only the most relevant is shown (see Fig. S2 to S10 for the remaining combinations).

The models were validated running 3 chains (no major differences were found between these), using the Gelman and Rubin diagnostic ([7]; R̂ was always between 1 and 1.02), inspecting the effective number of independent samples from the posterior, and performing posterior predictive checks. In both steps described above we used the Akaike weight based on the Widely Applicable Information Criterion (WAIC) of each model [8] to identify those models that are important. In all 36 models (eight regular models for all combinations of the three predictors, plus one model to determine the random structure, for each of the four response variables) the chains were stable, the posteriors were uncorrelated, and each model was able to predict the data.

References

1. Stephan JG, Lamei S, Pettis JS, Riesbeck K, de Miranda JR, Forsgren E. Honeybee-Specific Lactic Acid Bacterium Supplements Have No Effect on American Foulbrood-Infected Honeybee Colonies. Appl Environ Microbiol. 2019;85:1–12. doi:10.1128/AEM.00606-19.

2. Kruschke JK, Aguinis H, Joo H. The Time Has Come: Bayesian Methods for Data Analysis in the Organizational Sciences. Organ Res Methods. 2012;15:722–52.

3. McElreath R. Statistical Rethinking: A Bayesian Course With Examples in R and Stan. 2015. doi:10.3102/1076998616659752.

4. Kruschke JK. Doing Bayesian data analysis: A tutorial with R, JAGS, and Stan, second edition. 2nd edition. Elsevier Inc.; 2014. doi:10.1016/B978-0-12-405888-0.09999-2.

5. Schielzeth H. Simple means to improve the interpretability of regression coefficients. Methods Ecol Evol. 2010;1:103–13.

6. Harrison XA. Using observation-level random effects to model overdispersion in count data in ecology and evolution. PeerJ. 2014;2:e616. doi:10.7717/peerj.616.

7. Gelman A, Rubin DB. Inference from Iterative Simulation Using Multiple Sequences. Stat Sci. 1992;7:457–72. doi:10.1214/ss/1177011136.

8. Hooten MB, Hobbs NT, Ellison AM. A guide to Bayesian model selection for ecologists. Ecol Monogr. 2015;85:3–28.
